# The lunar core can be a major reservoir for volatile elements S, Se, Te and Sb

**DOI:** 10.1038/s41598-017-15203-0

**Published:** 2017-11-06

**Authors:** Edgar S. Steenstra, Yanhao Lin, Dian Dankers, Nachiketa Rai, Jasper Berndt, Sergei Matveev, Wim van Westrenen

**Affiliations:** 1 Faculty of Sciences, VU Amsterdam, De Boelelaan 1085, 1081 HV Amsterdam, The Netherlands; 2 Department of Earth and Planetary Sciences, Birkbeck University of London, London, UK; 3 Department of Earth Sciences, Mineral and Planetary Sciences Division, Natural History Museum, London, UK; 4 Institute of Mineralogy, University of Münster, Münster, Germany; 5 Faculty of Geosciences, Utrecht University, Utrecht, The Netherlands

## Abstract

The Moon bears a striking compositional and isotopic resemblance to the bulk silicate Earth (BSE) for many elements, but is considered highly depleted in many volatile elements compared to BSE due to high-temperature volatile loss from Moon-forming materials in the Moon-forming giant impact and/or due to evaporative loss during subsequent magmatism on the Moon. Here, we use high-pressure metal-silicate partitioning experiments to show that the observed low concentrations of volatile elements sulfur (S), selenium (Se), tellurium (Te), and antimony (Sb) in the silicate Moon can instead reflect core-mantle equilibration in a largely to fully molten Moon. When incorporating the core as a reservoir for these elements, their bulk Moon concentrations are similar to those in the present-day bulk silicate Earth. This suggests that Moon formation was not accompanied by major loss of S, Se, Te, Sb from Moon-forming materials, consistent with recent indications from lunar carbon and S isotopic compositions of primitive lunar materials. This is in marked contrast with the losses of other volatile elements (e.g., K, Zn) during the Moon-forming event. This discrepancy may be related to distinctly different cosmochemical behavior of S, Se, Te and Sb within the proto-lunar disk, which is as of yet virtually unconstrained.

## Introduction

It is now widely accepted that the Moon has an Fe-Ni core based on geophysical and geochemical considerations^1–7^, and the abundance patterns of many siderophile (iron-loving) elements in the lunar crust and mantle suggest this core formed in equilibrium with the silicate Moon^3–7^. Current lunar formation models suggest a very hot start for the materials forming the Moon, and assume this resulted in strong losses in volatile elements, leading to the depletion of many volatiles in the Moon compared to the bulk silicate Earth (e.g., refs^8–10^ and references therein). However, recent studies that focused on a wide range of primitive lunar samples have found that the Moon may be unexpectedly rich in highly volatile elements including hydrogen and carbon^11–13^. Some volatile elements, including sulfur (S), selenium (Se), tellurium (Te), and antimony (Sb), are both volatile and iron-loving (siderophile). These elements are currently assumed to be depleted in the lunar mantle purely as a result of temperature-induced evaporation or incomplete condensation during the Moon forming event^8,14^. The possibility that their siderophile behavior during lunar core-mantle differentiation could have resulted in preferential partitioning of these elements into the lunar core has not been considered. A lack of metal-silicate partition coefficients for these elements at lunar-relevant pressure and temperature conditions to date precluded an adequate assessment of their behavior during core formation.

## Results

### High pressure experiments

We obtained new experimental data for S, Se, Te, and Sb partitioning between (Fe,Ni) metal, FeS sulfide and silicate melts (Methods summary) to complement existing data focused on their behavior in the Earth^15–17^. A total of 33 experiments using synthetic metal-silicate mixtures doped with the four volatile elements were performed in a piston cylinder press using MgO capsules at temperatures between 1500 to 1600 °C and pressures from 1 to 2.5 GPa, under redox conditions of 2 to 1 log units below the iron-wüstite buffer (ΔIW) using two silicate melt compositions (Supplementary Information). Run products show segregated metallic blobs within a quenched silicate melt. Major and trace element concentrations were measured using electron microprobe and laser ablation-inductively coupled plasma – mass spectrometry (LA-ICP-MS) methods.

### Metal-silicate and sulfide-silicate partitioning of S, Se, Te, Sb

**Figure 1:**
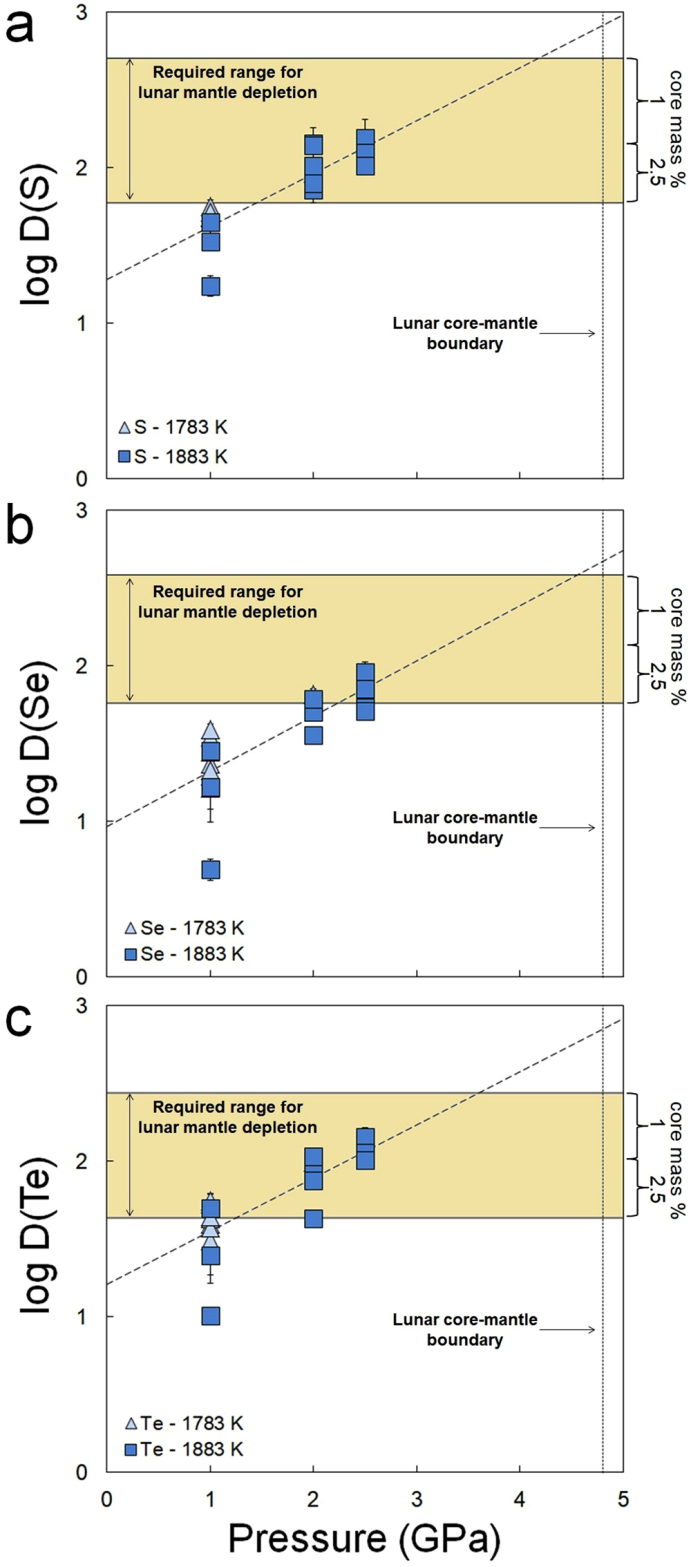
Metal-silicate partition coefficients (D) for S, Se, and Te for low S systems (<4 wt%) as a function of *P* (in GPa), normalized to 11 wt% FeO in the lunar mantle, corresponding to ΔIW = −2. Errors represent 1SE for LA-ICP-MS and 2SE for EMP measurements and were calculated using simple error propagation. Trend lines represent the derived pressure dependencies for D(S, Se, Te) (Supplementary Information). Horizontal bar represents the required metal-silicate partition coefficients to explain their lunar mantle depletions based on (refs^11,21,22^) for a 1 to 2.5 mass % lunar core. Lunar core-mantle equilibration depth is based on current geochemical and lunar formation models (refs^3,4,11,23,24^).

At constant *P* and *T*, a strong increase in metal affinity of S, Se, Te with decreasing FeO in the silicate melt is observed, which can be explained by the associated decrease of FeO(melt) activity^15,16^ (Supplementary Information). After normalization of the metal-silicate partition coefficients of S, Se, and Te to the FeO content of the lunar mantle of ~11 wt%^3^, we observe that sulfur enhances the siderophile behavior of Se and Te, consistent with their known chalcophile tendencies. In contrast, assuming 3+ as the dominant valence state of Sb in the silicate melt^17^, the preferential partitioning of Sb in metal is found to decrease significantly with increasing S in the metal. The siderophile behavior of S, Se, and Te increases dramatically with increasing pressure (Fig. 1). A comparison between our data and previous work^15^ performed at higher temperatures shows that temperature does not significantly affect their metal-silicate partitioning (Supplementary Information). Based on four experiments conducted in graphite saturated conditions, a strong decrease in the siderophile behavior of Sb with increasing temperature was previously proposed^17^. However, our results show no significant difference with their reported higher temperature metal-silicate partition coefficients. We also observe no pressure dependency on the metal-silicate partitioning behavior of Sb.

### Expected depletions of S, Se, Te and Sb due to lunar core formation

Our new data, covering pressure-temperature conditions and bulk compositions directly relevant to the lunar interior, were used to asses to what extent the estimated lunar mantle depletions of S, Se, Te, and Sb can be explained through segregation to a Fe-rich metal alloy lunar core by using equation (1) ^3–5^:

1 $${D}_{\frac{c(i)}{m(i)}}=\,\frac{{C}_{{\rm{BM}}({\rm{i}})}\,-\,x{C}_{{\rm{BSM}}({\rm{i}})}}{{C}_{{\rm{BSM}}}(i)(1\,-\,x)}$$

where C_BM(i)_ is the concentration by weight of element *i* in the bulk Moon, C_BSM(i)_ is the concentration by weight of element *i* in the bulk silicate Moon (BSM) and *x* is the mass fraction of the lunar mantle, assumed here to lie between 0.975–0.990^1,3–5^. To assess the maximum effect of core formation on bulk silicate Moon volatile element abundances, bulk Moon initial concentrations were considered to be equal to bulk silicate Earth (BSE) values^18–22^ (Table 1) (Supplementary Information).

**Table 1:** Bulk Moon and bulk silicate Moon abundances of S, Se, Te, and Sb and corresponding metal-silicate partition coefficients (D) to explain their depletion in the lunar mantle for a 2.5 mass% lunar core^3–5^ corrected for different late veneer scenarios.

	bulk Moon (BM)^21,22^	bulk silicate Moon (BSM)^11,26,53^	required log D^*^	BSM - 0.05 mass % H^19,22^	corrected log D	BSM - 0.05 mass % CM2^19,22,28^	corrected log D
S (ppm)	250 ± 50	74.5 ± 4.5	1.96 ± 0.20	63.5 ± 4.5	2.06 ± 0.08	58.3 ± 4.5	2.11 ± 0.07
Se (ppb)	80 ± 17	24	1.95 ± 0.19	19.9 ± 0.7	2.08 ± 0.11	17.3 ± 0.4	2.15 ± 0.11
Te (ppb)	11 ± 1.7	4.1	1.83 ± 0.19	3.90 ± 0.05	1.86 ± 0.10	3.33 ± 0.03	1.96 ± 0.09
Sb (ppb)	5.5	0.078	3.44 ± 0.34	0.043**	3.71	0.020	4.04
	BSE	BSM	BSM − 0.05 mass % H	BSM − 0.05 mass % CM2			
S/Se	2600 ± 700	3100 ± 190	3760 ± 360	4310 ± 360			
Se/Te	7.9 ± 1.6	5.9 ± 1.2***	5.1 ± 0.2	6.0 ± 0.3			

*Assuming a ± 10% uncertainty on log D values.

**Assuming CM estimate instead of CM2 due to lack of data.

***Assuming a ± 20% uncertainty as similarly reported for BSE ratio.

**Figure 2:**
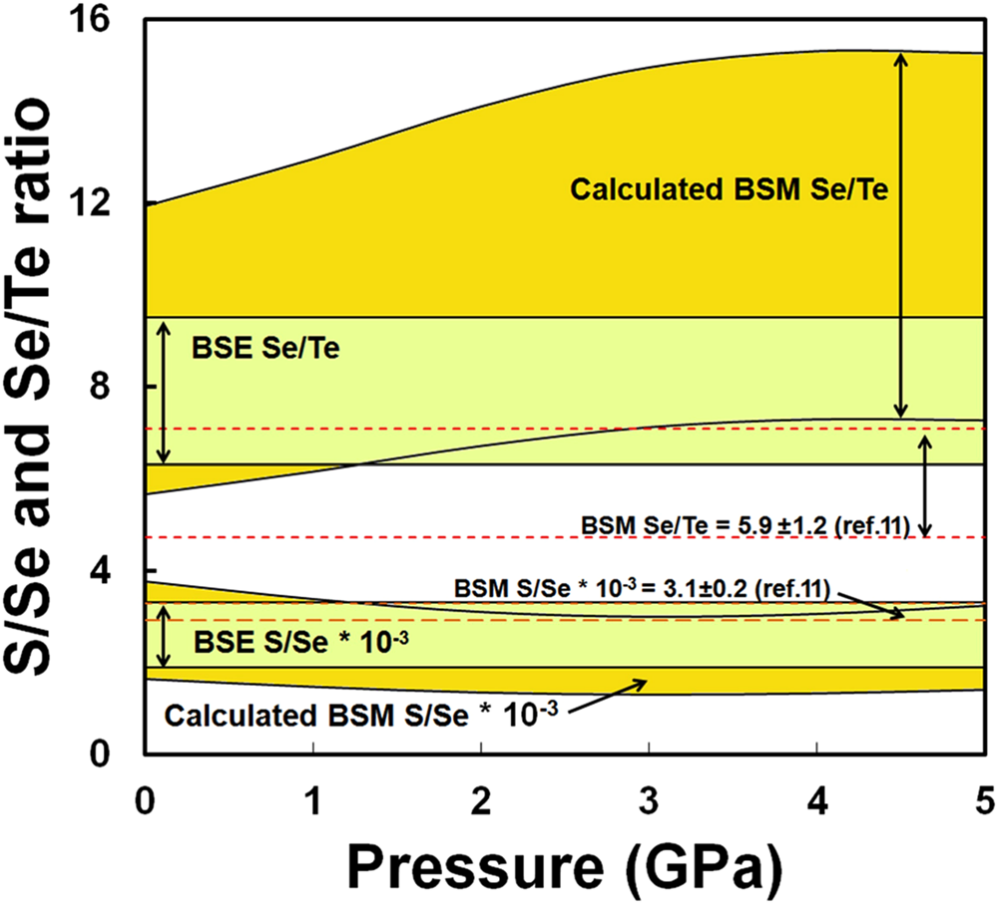
Calculated bulk silicate Moon (BSM) S/Se and Se/Te ratios as a function of different pressures during lunar core-mantle equilibration assuming a 2.5% lunar core mass. Also included are the S/Se and Se/Te ratios inferred for the bulk silicate Earth^22^ (BSE) and BSM estimates derived from ref.^11^.

Bulk silicate Moon abundances were taken from a recent compilation of lunar sample analyses^11^. These lunar mantle compositional estimates are partly based on the lunar volcanic glasses, for which there is controversy if these glasses are truly representative for the primitive lunar mantle (Supplementary Information). However, given their primitive nature in terms of Mg#, lower CaO and Al_2_O_3_, these glasses should provide a reasonably accurate reflection of the primitive lunar mantle, at least for the purpose of this study^11^. Using equation 1, we calculate which metal-silicate partition coefficients of S, Se, Te, and Sb would be required to fully explain their lunar mantle abundances by metal-silicate equilibration during core formation (Table 1). Assuming the average estimate of lunar mantle FeO (~11 wt%), corresponding to ~ΔIW = −2 (refs^3–5^), we observe that the lunar mantle depletions of S, Se, and Te are easily reconciled with depletion through core formation if equilibration occurred at high pressures (>2–3 GPa) in the Moon (Fig. 1). The pressure of equilibration is dependent of the assumed lunar core mass and increases with decreasing core size to fully molten conditions for a ~1% lunar core mass. Metal-silicate equilibration at high pressures (implying core formation in a largely or completely molten Moon) is also consistent with the observed abundances of fifteen other siderophile elements^4^ and with the calculated thermal consequences of giant impact-based lunar formation models^23,24^. These results can also be reconciled with recent experimental results of lunar magma ocean crystallization if this ocean contained slightly more water than that estimated for an intermediate depth lunar magma ocean, or if Al_2_O_3_ contents of the BSE during formation of the Moon are slightly overestimated. Figure 2 shows the effect of pressure on the resulting S/Se and Se/Te ratios of the BSM. These ratios are close or within error of the inferred BSE ratios^21,22^ at the conditions at which their lunar mantle elemental abundances are also explained by core formation depletion.

**Figure 3:**
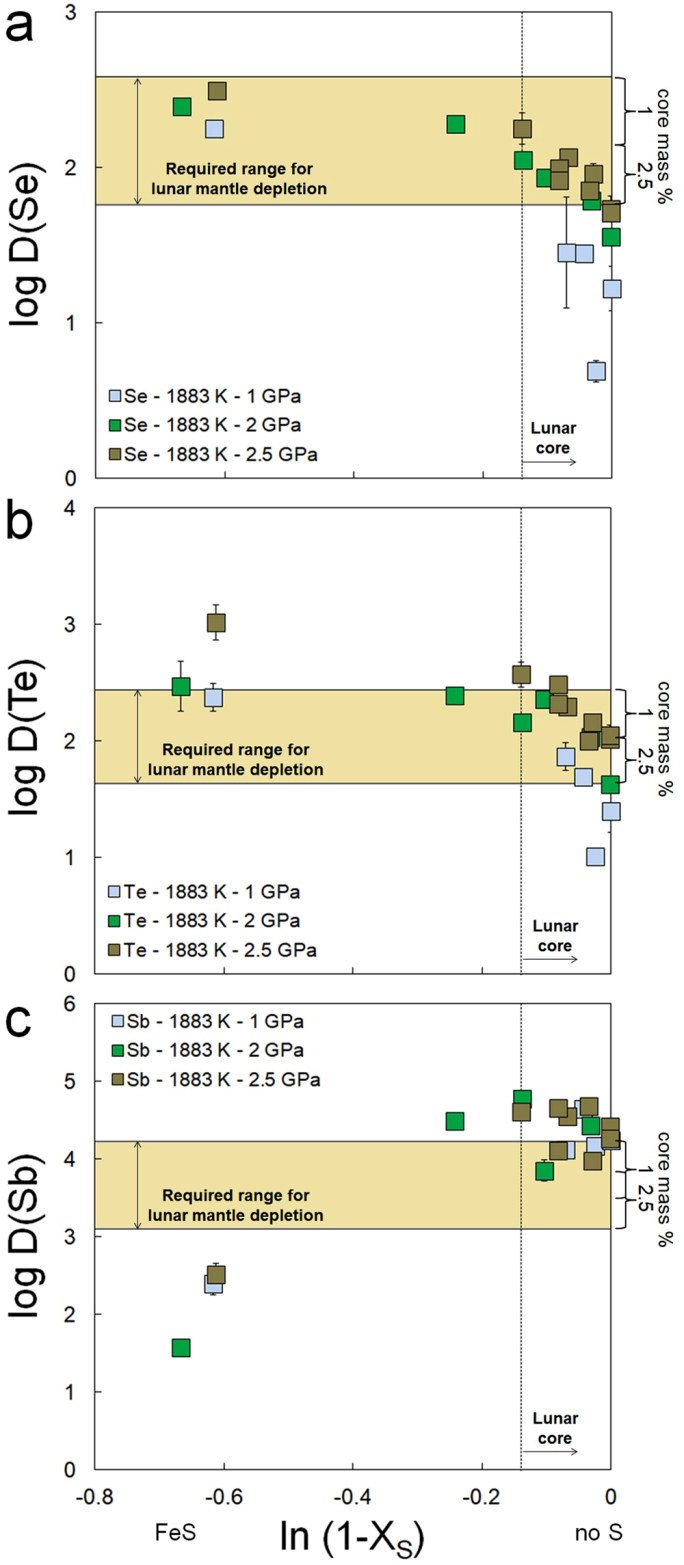
Metal-silicate partition coefficients (D) of Se, Te and Sb as a function of S content of the metal (defined as the natural logarithm of one minus the molar fraction of S in the metal) at constant *P* and *T* (1600 °C), corrected for different FeO contents of the silicate melt. Errors represent 1SE for LA-ICP-MS and 2SE for EMP measurements and were calculated using simple error propagation. Horizontal bar represents the required metal-silicate partition coefficients of Se, Te, and Sb to explain their lunar mantle depletions based on (refs^11,21,22^) for a 1 to 2.5 mass% lunar core. Vertical dotted line represents the maximum sulfur content of the lunar core from Laneuville *et al*. (ref.^25^).

The nature and abundance of one or more elements lighter than iron in the lunar core should also be taken into account, as this may drastically affect the metal-silicate partitioning of the elements considered here. Recent studies suggest that the S budget of the lunar core ranges anywhere between ~0–8 wt%^3–5,25^. For Se and Te, addition of S to the lunar core would further increase the feasibility of explaining their low mantle abundances by segregation into the core, as S significantly increases their siderophile behavior (Fig. 3). On the contrary, Sb shows chalcophobic behavior resulting in a significant decrease with S in the metal (Fig. 3). The estimated range of the lunar core S budget is not high enough to sufficiently reduce the siderophile behavior of Sb at ~ΔIW = −2. Steenstra *et al*. (ref.^4^) recently showed that the lunar mantle depletions of fifteen refractory and volatile siderophile elements do not necessarily require a S-bearing core. The lunar mantle depletion of Sb is therefore also consistent with core formation depletion only. Given the similar dependency of D(Se, Te) on metallic sulfur content, combined with the limited S content of the lunar core (<8 wt%), significant fractionations between Se and Te are not expected.

## Discussion

### The lunar core as a significant reservoir for volatile elements?

Our results demonstrate that the lunar core can be a significant reservoir for volatile elements S. Se. Te and Sb, consistent with previous observations for a suite of other volatile elements (C, Pb, In, Ga, Cu, Sn, Cd)^4,5^. The measured lunar mantle depletions of four (highly) volatile siderophile elements with a range of condensation temperatures and geochemical behavior, can be explained fully by their preferential partitioning into the metallic core during lunar metal-silicate segregation, even when the starting composition of the Moon is set equal to the composition of the BSE (Figs 1, 3). Sulfide saturation in the source area of lunar volcanic samples that are used to estimate BSM abundances could have lowered the lunar mantle abundances as well, but analyses of primitive low-Ti basalts, which show no evidence for sulfide saturation^26^, do not lead to different BSM concentration estimates. Sulfide saturation in the lunar mantle seems unlikely given the near-chondritic HSE patterns of many primitive lunar basalts^27^ (Supplementary Information).

### Effects of late accretion on S, Se, Te and Sb systematics

Abundances of highly siderophile elements (HSE) in the lunar mantle have been used to argue for the delivery of 0.02 to 0.05 mass wt% of accreted chondritic material shortly after the onset of lunar magma ocean crystallization, consistent with tungsten isotopic evidence^19,27^. The maximum addition of 0.05 mass wt% of two extreme chondritic end members (H and CM2 chondrites)^19,27,28^ to the bulk silicate Moon, assuming no volatile loss during impact, does not significantly affect the elemental abundances of S, Se, Te and/or Sb nor the Se/Te ratio of the BSM (Table 1; Figs 2, 3). With increasing addition of late veneer mass, the S/Se ratio increases but remains close to the BSE ratio (~2600 ± 700), except for the case of 0.05 mass wt% added CM2 material. The latter results likely reflects that the amount or nature of CM2 material is not realistic for the Moon, as the S/Se ratio is strongly dissimilar of the S/Se ratio of any other meteorite group or planetesimals.

Figure 1 shows that there is a considerable over-depletion of S, Se, Te for larger lunar core masses if the Moon was fully molten when its core formed. This would imply that S, Se and Te had to be delivered in significant quantities to the bulk silicate Moon after lunar core formation. Using the differences between the required D values and experimental D values, we calculate the amount of required added mass to be ~0.28 mass% for S, 0.23% for Se, and 1% for Te^19,27,28^ when assuming a H chondrite dominated late veneer. A CM2 chondritic late veneer yields lower masses, from ~0.14 mass% for Se to 0.19 and 0.24 mass% for S and Te. All of these values are much higher than the maximum veneer indicated by HSE abundances in the lunar mantle^27^. We conclude that for small lunar core masses, no late veneer is required, independent of magma ocean depth. For larger core masses, the observed differences between predicted and required D’s at greater magma ocean depths cannot be explained by a late veneer, but rather imply the formation of a lunar core at intermediate magma ocean pressures.

Comparison of the near chondritic measured Se/Te (5.9 ± 1.2)^11^ and/or calculated Se/Te ratio of the silicate Moon (Fig. 1) with the BSE ratio (7.9 ± 1.6)^22^ yields a striking similarity between the two, virtually independent of the amount or composition of added late veneer mass that is required to satisfy HSE depletions^19,27^ (Table 1), or the pressure of lunar core-mantle equilibration. Similarly, the measured S/Se and calculated S/Se ratios of the silicate Moon are largely within error with that of the BSE. We also note that a volatile-rich veneer addition to the BSE may not be required for S^16^, although this is still a matter of great debate^29,30^. This is mainly due to the fact that high-pressure data for S directly relevant for terrestrial core formation is scarce and that the outcome of these models heavily depends on the various core formation scenarios considered. Note that the chondritic nature of the BSE Se/Te ratio also remains heavily debated^16,22,31–33^.

### Extent and timing of volatile loss in the Earth-Moon system

Despite uncertainties in indigenous Se, Te and Sb abundances in the lunar mantle and the actual lunar core mass, the excellent agreement between the required metal-silicate partition coefficients and their experimentally determined partition coefficients, combined with the Se/Te ratio of the lunar mantle, strongly suggests that the BSM abundances of S, Se, Te, and Sb represent metal-silicate equilibration upon lunar core formation. Limited volatile loss during the Moon-forming event is in good agreement with the inferred early wet start of the Moon from experimental crystallization of the lunar magma ocean^13^. It also agrees with the observation that the lunar mantle depletion of C can be explained by high temperature lunar core formation^5^. Our results are also consistent with the S isotopic fractionation measured in primitive lunar mare basalts, that limits bulk Moon S degassing to less than 10% during the Moon-forming event^34^. Note that the heavier isotopic signature of S in lunar rocks may also be reconciled with preferential partitioning of S in the lunar core, as this would produce S isotopic fractionations of a similar magnitude^35^.

However, several other key volatile elements (K, Rb, Ga, Zn, Cl) show fractionations in lunar silicate materials, relative to their isotopic composition in bulk silicate Earth^9,36–44^. This implies that these elements were partly lost during the Moon-forming event. Besides evaporative loss during lunar formation, other mechanisms have been proposed to explain isotopic fractionations of these elements in lunar samples. The large isotopic fractionation of Cl is thought to be related to its volatilization as a metal halide during the lunar magma ocean (LMO) stage or due to its loss during basalt eruptions on the lunar surface, rather than evaporation during the Moon-forming event^38–40^. Current isotopic fractionation models for most other volatile elements cannot distinguish whether their heavy isotopic compositions lunar silicate materials were set during the Moon-forming event and/or during a later global magmatic event, such as crystallization of the LMO^9,41–43^. Our results imply minor or no loss of S, Se, Te, and Sb during the Moon-forming event, which could suggest that the isotopic fractionations of other moderately volatile elements may have occurred later in lunar history, rather than during the Moon-forming event. This is because S, Se, Te are more volatile than elements K, Rb, Ga, and similarly volatile as Zn. However, the significant depletions and isotopic fractionations of relatively heavy elements K, Rb and Zn cannot be reconciled with LMO degassing only. It seems indeed inevitable that a significant fraction of these latter elements were lost during the Moon-forming event^37,43,44^. The discrepancy between these observations and our results for S, Se, Te and Sb, as well as the isotopic results for S^35^, may reflect significantly different geochemical behavior of S, Se, Te and Sb during the Moon-forming event^45^.

We do note that some Zn and Ga loss may be attributed to their preferential partitioning into the lunar core. For example, recent experimental studies on Zn metal-silicate partitioning suggest that temperature drastically increases the siderophile tendencies of Zn, whereas Ga behaves as a moderately siderophile element under a wide range of lunar core formation conditions^31,46–48^. The possible extent of partitioning of Zn, Ga and other volatile elements into the lunar core should therefore be addressed in future work. Nevertheless, the isotopic fractionations of Zn and Ga still point to significant evaporative loss during the Moon-forming event.

We conclude that the preferential sequestering of volatile elements S, Se, Te and Sb in the lunar core is a natural consequence of lunar differentiation at the conditions predicted from independent lunar formation and evolution models. Our results suggests that Earth obtained most of its S-Se-Te-Sb volatile budget before the Moon-forming event, and that the Moon inherited this budget without significant loss of these elements during its formation. Our results show that the lunar core may in fact be a significant “hidden” reservoir for several volatile elements, which should be taken into account for future models focused on lunar volatile evolution.

## Methods

### High pressure experiments

Experiments were performed at 1500 to 1600 °C from 1 to 2.5 GPa in a Bristol-type end-loaded piston-cylinder press at Vrije Universiteit Amsterdam^49^. Metal-silicate and sulfide-silicate partitioning experiments were performed using MgO capsules manufactured from high-purity polycrystalline rods that were placed within a half-inch diameter talc-pyrex cell assembly^50^. Starting compositions consisted of a synthetic analogue of the lunar Apollo 15 green glass or a typical lunar granitic composition (Supplementary Tables 1, 2). Variable amounts of FeS and approximately 1 wt.% Se, Te, and Sb were added as pure metals in addition to other trace elements (Supplementary Table 1). Temperature was monitored using a W_97_-Re_3_-W_75_Re_25_ (type D) thermocouple and Eurotherm 2404 programmable controller. The center of the samples was located in the hotspot of the assembly, 2 mm away from the thermocouple tip, so that sample temperatures were within 10 °C of the thermocouple reading^51^. Pressure was gradually increased during heating (hot piston-in technique). Experiments were rapidly quenched by shutting off the power to the furnace. Recovered samples were mounted in petropoxy resin, section perpendicular to the capsule and polished to a fine (<1 µm) for electron microprobe (EMP) and LA-ICP-MS analyses. A time series between 15 and 120 minutes was performed to ensure that metal-silicate equilibrium was attained (Supplementary Information).

### Analytical techniques

After samples were carbon-coated, major element abundances in the silicate and metal were measured using a JEOL JXA-8800M Electron Microprobe at Utrecht University (Supplementary Tables 4, 5). Analysis was done using an accelerating voltage of 15 kV. A 5 µm sized beam was used for homogeneous phases and a 15 µm diameter beam for heterogeneous phases. Metal standards for electron microprobe analyses consisted of tephroite for Mn, chalcopyrite for S, galena for Pb, InAs for In and As, CdS for Cd, and pure metal standards for Cr, Fe, Ni, Se, Sn, Sb, and Te. Silicate analyses were calibrated with diopside for Si and Ca, forsterite for Mg, corundum for Al, hematite for Fe, tephroite for Mn, KTiPO5 for P and K, TiO for Ti, jadeite for Na, chalcopyrite for S, galena for Pb, InAs for In, CdS for Cd and pure metal standards for Cr, Ni, Se, Sn, Sb, and Te.

Data was processed using the ZAF algorithm^52^. Laser ablation inductively coupled plasma mass spectrometry (LA-ICP-MS) was used to quantify the abundances of Se, Te, and Sb and other trace elements in the silicate melt (Supplementary Table 4). We used a 193 nm ArF excimer laser (Analyte G2, Photon Machines) laser with a repetition rate of 10 Hz and energy of ∼3-4 J/cm^2^ throughout the entire session with beam sizes ranging between 25-50 µm. We measured the following isotopes: ^29^Si, ^43^Ca, ^47^Ti, ^48^Ti, ^53^Cr, ^55^Mn, ^60^Ni, ^61^Ni, ^63^Cu, ^66^Zn, ^75^As, ^82^Se, ^93^Nb, ^111^Cd, ^115^In, ^118^Sn, ^121^Sb, ^125^Te, ^181^Ta and ^208^Pb. The NIST 612 glass was used as an external reference material for both the metal and silicate. Previously determined ^29^Si (silicates) and ^60^Ni (metals) values measured by electron microprobe were used as internal standards. We observe that LA-ICP-MS analyses of Sb and other trace elements in USGS BIR-1G and BCR-2G standards are reproduced well compared to previously reported values analyses (Supplementary Information).

### Data availability

The datasets generated during and/or analysed during the current study are available from the corresponding author on reasonable request.

## Electronic supplementary material

Supplementary Information file

## References

[1] Williams JG (2014). Lunar interior properties from the GRAIL mission. J.Geophys. Res.: Planets.

[2] Weber RC, Lin P-Y, Garnero EJ, Williams Q, Lognonné P (2011). Seismic detection of the lunar core. Science.

[3] Rai N, van Westrenen W (2014). Lunar core formation: new constraints from metal-silicate partitioning of siderophile elements. Earth Planet. Sci. Lett..

[4] Steenstra ES, Rai N, Knibbe JS, Lin YH, van Westrenen W (2016). New geochemical models of core formation in the Moon from metal-silicate partitioning of 15 siderophile elements. Earth Planet. Sci. Lett..

[5] Steenstra ES, Lin YH, Rai N, Jansen M, van Westrenen W (2017). Carbon as the dominant light element in the lunar core. Am. Mineral..

[6] Cartier C, Hammouda T, Boyet M, Bouhifd MA, Devidal J-L (2014). Redox control of the fractionation of niobium and tantalum during planetary accretion and core formation. Nat. Geosci..

[7] Elardo SM, Shahar A (2017). Non-chondritic iron isotope ratios in planetary mantles as a result of core formation. Nat. Geosci..

[8] Taylor GJ, Wieczorek MA (2014). Lunar bulk chemical composition: a post-Gravity Recovery and Interior Laboratory reassessment. Phil. Trans. R. Soc. A.

[9] Wang K, Jacobsen SB (2016). Potassium isotopic evidence for a high-energy giant impact origin of the Moon. Nature.

[10] Canup RM, Visscher C, Salmon J (2015). Bruce Fegley, Jr. Lunar volatile depletion due to incomplete accretion within an impact-generated disk. Nat. Geosci..

[11] Hauri EH, Saal AE, Rutherford MJ, van Orman JA (2015). Water in the Moon’s interior: truth and consequences. Earth Planet. Sci. Lett..

[12] Wetzel DT, Hauri EH, Saal AE, Rutherford MJ (2015). Carbon content and degassing history of the lunar volcanic glasses. Nat. Geosci..

[13] Lin YH, Tronche EJ, Steenstra ES, van Westrenen W (2017). Evidence for an early wet Moon from experimental crystallization of the lunar magma ocean. Nat. Geosci..

[14] Taylor SR (1987). The unique lunar composition and its bearing on the origin of the Moon. Geochim. Cosmochim. Acta.

[15] Rose-Weston L, Brenan JM, Fei Y, Secco RA, Frost DJ (2009). Effect of pressure, temperature, and oxygen fugacity on the metal-silicate partitioning of Te, Se, and S: Implications for earth differentiation. Geochim. Cosmochim. Acta.

[16] Boujibar A (2014). Metal–silicate partitioning of sulphur, new experimental and thermodynamic constraints on planetary accretion. Earth Planet. Sci. Lett..

[17] Righter K (2009). Experimental studies of metal-silicate partitioning of Sb: Implications for the terrestrial and lunar mantles. Geochim. Cosmochim. Acta.

[18] Zhang J, Dauphas N, Davis AM, Leya I, Fedkin A (2012). The proto-Earth as a significant source of lunar material. Nat. Geosci..

[19] Kruijer TS, Kleine T, Fischer-Gödde M, Sprung P (2015). Lunar tungsten isotopic evidence for the late veneer. Nature.

[20] Young ED (2016). Oxygen isotopic evidence for vigorous mixing during the Moon-forming giant impact. Science.

[21] McDonough WF, Sun SS (1995). The composition of the Earth. Chem. Geol..

[22] Wang Z, Becker H (2013). Ratios of S, Se and Te in the silicate Earth require a volatile-rich late veneer. Nature.

[23] Canup RM (2012). Forming a Moon with an Earth-like Composition via a Giant Impact. Science.

[24] Lock SJ (2016). A new model for lunar origin: equilibration with Earth beyond the hot spin stability limit. Lunar Planet. Sci. Conf..

[25] Laneuville M (2014). A long-lived lunar dynamo powered by core crystallization. Earth Planet. Sci. Lett..

[26] Bombardieri DJ, Norman MD, Kamenetsky VS, Danyushevsky LV (2005). Major element and primary sulfur concentrations in Apollo 12 mare basalts: The view from melt inclusions. Meteorit. Planet. Sci..

[27] Day JM, Walker RJ (2015). Highly siderophile element depletion in the Moon. Earth Planet. Sci. Lett..

[28] Newsom, H. E. Composition of the Solar System, Planets, Meteorites and Major Terrestrial Reservoirs. In: *Global Earth Physics*, *A Handbook of Physical Constants*, AGU Reference Shelf1.

[29] Ballhaus C (2017). The great sulfur depletion of Earth’s mantle is not a signature of mantle-core equilibration. Contrib. Min. Petrol..

[30] Suer TA, Siebert J, Laurent R, Menguy N, Guillaume F (2017). A sulfur-poor terrestrial core inferred from metal-silicate partitioning experiments. Earth Planet. Sci. Lett..

[31] Ballhaus C (2013). The U/Pb ratio of the Earth’s mantle – A signature of late volatile addition. Earth Planet. Sci. Lett..

[32] König S, Lorand J-P, Luguet A, Pearson DG (2014). A non-primitive origin of near-chondritic S-Se-Te ratios in mantle peridotites; implications for the Earth’s late accretionary history. Earth Planet. Sci. Lett..

[33] Brasser R, Mojzsis SJ, Werner SC, Matsumura S, Ida S (2016). Late veneer and late accretion to the terrestrial planets. Earth Planet. Sci. Lett..

[34] Wing BA, Farquhar J (2015). Sulfur isotope homogeneity of lunar mare basalts. Geochim. Cosmochim. Acta.

[35] Labidi J (2016). Experimentally determined sulfur isotope fractionation between metal and silicate and implications for planetary differentiation. Geochim. Cosmochim. Acta.

[36] Day JMD, Moynier F (2014). Evaporative fractionation of volatile stable isotopes and their bearing on the origin of theMoon. Phi. Trans. R. Soc. A..

[37] Paniello RC, Day JMD, Moynier F (2012). Zinc isotopic evidence for the origin of the Moon. Nature.

[38] Sharp ZD, Shearer CK, McKeegan KD, Barnes JD, Wang YG (2010). The chlorine isotope composition of the Moon and implications for an anhydrous mantle. Science.

[39] Barnes JJ (2016). Early degassing of lunar urKREEP by crust-breaching impact(s). Earth Planet. Sci. Lett..

[40] Boyce JW (2015). The chlorine isotope fingerprint of the lunar magma ocean. Sci. Adv..

[41] Kato C (2015). Extensive volatile loss during formation and differentiation of theMoon. Nat. Comm..

[42] Kato C, Moynier F (2017). Gallium isotopic evidence for extensive volatile loss from the Moon during its formation. Sci. Adv..

[43] Pringle EA, Moynier F (2017). Rubidium isotopic composition of the Earth, meteorites and the Moon: evidence for the origin of volatile loss during planetary accretion. Earth Planet. Sci. Lett..

[44] Day, J. M. D., Moynier, F., Shearer, C. K. Late-stage magmatic outgassing from a volatile-depleted Moon. *PNAS*, 10.1073/pnas.1708236114 (2017). 10.1073/pnas.1708236114 PMC5594690 28827322

[45] Ringwood AE, Kesson SE (1977). Basaltic magmatism and the bulk composition of the Moon. Earth, Moon, Planets.

[46] Mahan B, Siebert J, Pringle EA, Moynier F (2017). Elemental partitioning and isotopic fractionation of Zn between metal and silicate and geochemical estimation of the S content of the Earth’s core. Geochim. Cosmochim. Acta.

[47] Siebert J, Corgne A, Ryerson FJ (2011). Systematics of metal-silicate partitioning for many siderophile elements applied to Earth’s core formation. Geochim. Cosmochim. Acta.

[48] Blanchard I, Badro J, Siebert J, Ryerson FJ (2015). Composition of the core from gallium metal-silicate partitioning experiments. Earth Planet. Sci. Lett..

[49] Steenstra, E. S. *et al.* The effect of melt composition on metal-silicate partitioning of siderophile elements and constraints on core formation in the angrite parent body. *Geochim. Cosmochim. Acta* **212,** 62–83 (2017).

[50] van Kan Parker M, Mason PRD, van Westrenen W (2011). Experimental study of trace element partitioning between lunar orthopyroxene and anhydrous silicate melts: effects of lithium and iron. Chem. Geol..

[51] Watson EB, Wark D, Price JD, van Orman JA (2002). Mapping the thermal structure of solid-media pressure assemblies. Contrib. Mineral. Petrol..

[52] Reed, S. J. B. *Electron Microprobe Analysis And Scanning Electron Microscopy in Geology*. Cambridge University Press, Cambridge, UK (2005).

[53] Chen, Y. *et al*. Water, fluorine, and sulfur concentrations in the lunar mantle. *Earth Planet*. *Sci*. *Lett*. **427**, 37–46.

