# Supplementary material for: The lunar core can be a major reservoir for volatile elements S, Se, Te and Sb
Source: Sci Rep. 2017 Nov 6;7:14552. doi: 10.1038/s41598-017-15203-0 (PMC5673932; doi:10.1038/s41598-017-15203-0)
Supplement: Supplementary file 1 — Supplementary Information file [file 41598_2017_15203_MOESM1_ESM.pdf]

Supplementary Information file for ''The lunar core can be a major reservoir for volatile elements S, Se, Te and Sb'' by Edgar S. Steenstra, Yanhao Lin, Dian Dankers, Nachiketa Rai, Jasper Berndt, Sergei Matveev, Wim van Westrenen.

Experimental run products

Typical run products consisted of segregated metallic blobs within a quenched silicate melt. The silicate melt quenched to a spinifex textured glass (Fig. S1).

Verification of analytical precision of LA-ICP-MS analysis

USGS reference materials BIR-1G and BCR-2G were analyzed every ~20 LA-ICP-MS spots to assess the analytical accuracy and precision of measured trace element concentrations in the silicate melt. We observe that our analyses generally agree well with the GeoReM recommended value[54] (Fig. S2). We cannot compare for Se and Te, as they are below detection limit in the silicate reference materials. However, given that we also obtain concentrations below the detection limit, we expect no systematic offset. This is confirmed by comparison of the results obtained with electron microprobe (>500 ppm) with those derived using LA-ICP-MS (Fig. S3), showing excellent agreement. Given the lower detection limit of LA-ICP-MS, we use LA-ICP-MS data for all trace element measurements of the silicate melt. Because of the heterogeneous nature of the metal, we used electron microprobe analysis to quantify the abundance of major elements in the metals.

Assessment of possible effects of low totals of metals on D

Some of the analyzed metal blobs have porous textures, that likely resulted from decompression of this phase upon quenching. This resulted in lower EMPA totals for some of these phases (Table S5). Similarly consistently low totals for metal phases are found in several other published metal-silicate experiments performed in MgO capsules[55,56]. Note that this cannot be the result from dissolution of C in the metal, something which is seen in metals from experiments performed in graphite capsules. It could also be a result from a difference in EPMA calibration of Fe. Independent of the exact cause, given the relatively low abundances of the trace elements compared with the difference from 100%, it is evident that these issues will not significantly affect the outcome of this study. We considered the possible effects of the slightly lower totals on D. The table below reports the resulting differences for run GG0S-5 (which has the lowest total of the samples presented), suggesting our results are not affected (within error) by the lower totals.

Equilibrium

Previous studies have shown that equilibrium between metal and silicate is attained within <15 min in experiments that use similar capsule dimensions, *P* and *T*[55-57]. To test these observations, we performed a time series at 1783 K and 1 GPa with similar silicate and metal compositions. The FeO content of the silicate melt varied among the experiments and log D(S, Se, Te) were thus normalized to xFeO = 0.08 using dependencies discussed later in this section. We observe no significant difference in their D's across the time range of 15-120 min (Fig. S4). This implies that equilibrium is attained within <15 min at 1783 K. In one of the runs, Sb was below detection limit and equilibrium could therefore not be assessed for this element. However, Kiseeva and Wood (ref. 58) showed that equilibrium for Sb is already attained within 30 min at 1683 K. Given that diffusion rates increase substantially with increasing *T*, this implies Sb equilibrium was attained in our experiments.

Corrections for FeO and variable fO_2
Despite the use of only two silicate melt compositional end-members, there is still considerable variability in FeO (and therefore fO_2). The metal-silicate partitioning of S, Se, Te is clearly dependent on FeO content of the silicate melt, as previously observed[15.16] (Fig. S5). At low sulfur contents (X_S <0.04), T = 1783 K and P = 1 GPa, we derive the following slopes of log D(S, Se, Te) with FeO: -4.37±0.41 (R^2 = 0.97) for S, -5.16±0.95 (R^2 = 0.86) for Se, -4.18±0.63 (R^2 = 0.90) for Te. We compare these dependencies for recently reported metal-silicate partitioning data of Se and Te[57,58]. We observe that the slopes of D(Se, Te) versus molar fraction of FeO for C-saturated experiments at 2 GPa and 1683-1883 K show very similar slopes[59]. This is also the case for the 1 GPa data obtained in MgO capsules at 1883 K[60]. For Sb, we correct for variable fO_2 assuming its 3+ valence state in silicate melts[17].

Pressure dependency of D(S, Se, Te)
After correction for variable FeO contents, a distinct increase with pressure of D(S, Se, Te) is observed. Linear regression of all low S (X_S <0.04) data yields: log D (FeO corrected) = 0.34±0.04 *P (GPa) + 1.28±0.08 (R^2 = 0.76) for S, log D (FeO corrected) = 0.35±0.07 * P(GPa) + 0.97±0.12 (R^2 = 0.59) for Se, and log D (FeO corrected) = 0.34±0.06*P(GPa) + 1.21±0.11 (R^2 = 0.62) for Te. The calculated dependencies of D(S, Se, Te) on pressure are plotted as trend-lines in Fig. 1.

Temperature effects on D (S, Se, Te)
Temperature may affect the metal-silicate partitioning of S, Se, Te. Figure S6 shows the corrected D (S, Se, Te) values from Rose-Weston et al. (ref. 15) and our work as a function of temperature. All D values were corrected to a common reference pressure of 3 GPa and the molar FeO content of the lunar mantle, using the dependencies discussed earlier in this file. Only D values obtained at <5 GPa were considered, as the pressure dependency of S, Se and Te is likely significantly different >5 GPa relative to <5 GPa, as has been suggested for many other siderophile elements (e.g., ref. 60). In addition, only D values for silicate systems with FeO contents that were close to the lunar mantle were considered in the comparison, due to the highly non-ideal behavior of S, Se and Te at very low FeO contents[15,60]. Comparison of our new data with the data from (ref. 15) suggests that there is no significant effect of temperature on S, Se, Te metal-silicate partitioning between 1773 – 2300 K.

Bulk silicate Moon estimates
In this study, we assume the S, Se, Te and Sb abundances in the lunar mantle compiled in Hauri et al. (ref. 11). This estimate is based on a wide range of lunar lithologies, including the lunar volcanic glasses and mare basalts. However, some workers have argued that the lunar volcanic glasses may be a local anomaly[62,63]. This could result in incorrect bulk silicate Moon estimates for S, Se, Te and Sb that were used in this study. In addition, Walker et al. (ref. 64) found evidence for meteoritic contamination of the lunar volcanic glasses, which potentially could affect the presence of moderately siderophile elements too. On the other hand, many authors have emphasized on the petrological significance of the lunar volcanic glasses and their close approximation of the primitive lunar mantle[11,12,45,48,65-71].

It has been shown that the mare basalts can be produced by fractional crystallization of parent magmas that lie on mixing lines that connect the compositions of the various lunar picritic glasses[11]. This illustrates that the volcanic

glasses are unlikely to be a local anomaly, but that they are one of the most primitive magmas produced by melting and melt migration in the lunar mantle[11]. The volcanic glasses therefore provide important constraints on the abundance of volatiles, including S, Se, Te, Sb, in the lunar interior and should be taken into account for bulk silicate lunar compositional models. Note that ref. (48) reported similar results for Cl, S and Fl. The latter authors found that their concentrations in lunar melt inclusions in orange volcanic glasses and within a wider range of lunar mare basalts are very similar to one another. This provides additional evidence for the hypothesis that lunar volcanic glasses are not local anomalies in terms of volatile elements. Indeed, Hauri et al. (ref. 11) use the S/Dy ratio to obtain the S concentration of 79 ppm in the bulk silicate Moon. This estimate is corrected for the presence of volatiles on the exterior of the volcanic beads and therefore likely represent indigenous S abundances for the lunar volcanic glasses (see below). Other authors found near-identical concentrations for different primitive lunar lithologies. Chen et al. (ref. 48) proposed 70 ppm S to be in the bulk silicate Moon, while consideration of both melt inclusions in lunar basalts and volcanic glasses. Bombardieri et al. (ref. 49) obtain identical results (75 ppm S) when considering melt inclusions in Apollo 12 picritic basalts only.

However, there are also significant uncertainties related to estimating primitive compositions from the primitive lunar volcanic glasses, which should be acknowledged. One aspect is that the glass beads are characterized by a volatile-rich coating that is believed to have been a result from its deposition onto the beads during cooling of clouds of volcanic gas in which the glass beads quenched[72]. Hauri et al. (ref. 11) reconstruct the indigenous volatile abundances of these beads by assuming that the coating represents the actual volcanic gas. They measured the difference in S abundances in the least degassed melt inclusion in 74220 and ratio the abundance of all the other trace elements in 74220 in the surface coating to S. Indigenous abundances of volatile elements in 74220 were than calculated assuming the least-degassed S content of melt inclusions in 74220. Uncertainties in this approach arise due to the fact that the surface coating is unlikely to be a perfect estimate of the actual volcanic gas, but nevertheless likely reflects the best estimate currently available for pre-eruptive abundances of these elements (ref. 11). As previously mentioned, Walker et al. (ref. 64) found compelling evidence for meteoritic contamination of the volcanic glass beads based on HSE systematics. The latter workers found that the rims (or etchates) of the volcanic glass beads are characterized by (near)-chondritic relative HSE abundances (i.e. dominated by meteoritic sources), whereas the interior of the beads were inferred to be the indigenous component. It should be assessed to which extent these observations may affect our results for less siderophile elements. For this purpose, we use key element Ir as a proxy for the amount of material added to these volcanic beads and assume a H chondritic impactor. The largest offset of Ir between interiors (residues) and coatings (etchates) of the volcanic glasses in the study of Walker et al. (ref. 64) is 820 ppt Ir, but mostly scatters around <100 ppt. A typical H-chondrite contains 760 ppb Ir (ref. 28). This shows that approximately 0.0001% meteoritic material would need to be added in order to reproduce the Ir enrichment. In the case of a H-chondrite this would relate to 0.0001% of 7.7 ppm Se, 260 ppb Te, and 70 ppb Sb, or in absolute abundances, 0.00077 ppm Se (or 0.8 ppb Se), 0.03 ppb Te and 0.007 ppb Sb, assuming no devolatilization on impact and perfect mixing. Given the order of magnitude of the abundances inferred for the bulk silicate Moon, this will not significantly change the estimated abundances of these elements (see Table 1 in main manuscript).

Chemical similarity of bulk silicate Earth (BSE) and bulk Moon (BM)

Several major lines of evidence point to a highly similar composition of the bulk silicate Earth and bulk Moon. First, current ideas and dynamical models of lunar formation predict an identical or highly similar composition[23,24,73]. Another major line of evidence is the isotopic similarity between the BSE and the BM. Isotopic composition is a significant, genetically diagnostic aspect of bulk composition. All isotopic signatures of lunar rocks of all considered isotopic systems, except for W and possibly (but not likely) O, have found to be within error of the bulk silicate Earth (e.g., ref. 18-20). This is even the case for Cr and Ti, which manifest great diversity among planetary materials, but still are indistinguishable for the Earth and bulk Moon[74,75]. Consistent match-up among several of these many different isotope systems is implausible. The difference in W has been attributed to a late veneer of 0.05 mass %[19], so this difference is not related to true differences in BSE and BM. Therefore, all isotopic systems imply the BSE is the same as BM.

Another line of evidence is the similarity in abundances of major and trace elements in the BSE and BM. A wide variety of major elements of the BSE have been found to be very close or within error of that of BSE, which includes virtually the whole class of refractory lithophile elements (SiO_2, Al_2O_3, CaO, TiO_2 and 25 trace elements, including Cr, V, U, Th and REE[8,76-79]. The only major difference is the different Fe contents of the BSE and BM. However, ref. (77) suggests that temperature uncertainties for the lunar interior results in a significant uncertainty in FeO, and it may be closer to BSE then currently assumed. For example, ref. (79) concludes that FeO is only mildly enriched in the BSM. This is similarly the case for MgO[77]. A similar FeO content of the BSE and BM has also been recently inferred from Fe isotopic data of primitive lunar silicate materials (80).

Assessment of sulfide saturation of the lunar mantle

Sulfide saturation of the lunar mantle would result in the underestimation of the lunar mantle abundances of many siderophile elements that behave chalcophile (e.g., S, Se, Te. However, sulfur measurements on a wider range of lunar mare basalts suggest that their source region was never sulfide saturated[26,]. We also note that sulfur bulk lunar mantle estimate from these samples is within error with that of other lunar igneous rocks[11,26,45]. Additional evidence for the lack of sulfide saturation in the lunar mantle include the (1) near identical CI normalized HSE patterns for many lunar mare basalts relative to terrestrial systems (albeit lower)[81], (2) the highest MgO Apollo 12 lunar basalts have HSE abundances in nearly chondritic abundances[27] and the strong linear correlation between W and strongly incompatible lithophile trace elements U and W[27].

Supplementary References

54. Jochum, K. P. et al. GeoReM: a new geochemical database for reference materials and isotopic standards. *Geost. Geoanal. Res.* **29,** 333-338 (2005).

55. Tuff, J., Wood, B. J., Wade, J. The effect of Si on metal-silicate partitioning of siderophile elements and implications for the conditions of core formation. *Geochim. Cosmochim. Acta* **75,** 673-690 (2011).

56. Wade, J., Wood, B. J., Tuff, J. Metal-silicate partitioning of Mo and W at high pressures and temperatures: Evidence for late accretion of sulphur to the Earth. *Geochim. Cosmochim. Acta* **85,** 58-74 (2012).

57. Righter, K., Drake, M. J., Yaxley, G. Prediction of siderophile element metal-silicate partition coefficients to 20 GPa and 2800 °C: the effect of pressure, temperature, fO_2 and silicate and metallic melt composition. *Phys. Earth Planet. Inter*. **100**, 115-134 (1997).

58. Kiseeva, E. S., Wood, B. J. A simple model for chalcophile element partitioning between silicate and sulphide liquids with geochemical applications. *Earth Planet. Sci. Lett.* **383**, 68–81 (2013).
59. Crockett, M. J. et al. The effects of carbon on metal-silicate partitioning of volatile siderophile elements and core formation in the Moon. ***47th*** *Lunar Planet. Sci. Conf.*. #1054 (2017).
60. Seegers A. X. et al. The effects of Si and fO_2 on the metal-silicate partitioning of volatile siderophile elements: Implications for the Se/Te systematics of the bulk silicate Earth. ***47th*** *Lunar Planet. Sci. Conf.* #1050 (2017).
61. Kegler, P. et al. New Ni and Co metal-silicate partitioning data and their relevance for an early terrestrial magma ocean. *Earth Planet. Sci. Lett.* **268,** 28-40 (2008).
62. Albarede F., Albalat E., Lee C-T. A. An Intrinsic volatility scale relevant to the Earth and Moon and the status of water in the Moon. *Meteorit. Planet. Sci. 50,* 568-577.
63. Day, J. M. D., Moynier, F., Shearer, C. K. Late-stage magmatic outgassing from a volatile-depleted Moon. *PNAS,* doi:10.1073/pnas.1708236114 (2017).
64. Walker, R. J., Horan, M. F., Shearer, C. K., Papike, J. J. Low abundances of highly siderophile elements in the lunar mantle: evidence for prolonged late accretion. *Earth Planet. Sci. Lett.* **224,** 399-413 (2004).
65. Spudis P.D., Ryder G. Geology and petrology of the Apollo 15 landing site: Past, present and future understanding. *Eos* **66,** 721, 724-726 (1985).
66. Delano, J. W. Pristine lunar glasses: Criteria, Data and implications. *Proc. 16th Lunar Planet. Sci. Conf., J. Geophys. Res.* **91,** D201-D213 (1986).
67. Hughes *et al.* Apollo 15 yellow-brown volcanic glass: Chemistry and petrogenetic relations to green volcanic glass and olivine-normative mare basalts. *Geochim. Cosmochim. Acta* **52,** 2379-2391 (1988).
68. Jones J.H., Delano J.W. (1989) A three-component model for the bulk composition of the Moon. *Geochim. Cosmochim. Acta* **53,** 513-527
69. Shearer C.K., Papike, J.J. Basaltic magmatism on the Moon – a perspective from volcanic picritic glass beads. *Geochim. Cosmochim. Acta* **57,** 4785-4812 (1993).
70. Saal A.E. *et al.* Volatile content of lunar volcanic glasses and the presence of water in the Moon's interior. *Nature* **454,** 192-195 (2008).
71. Saal A.E. et al. Hydrogen isotopes in lunar volcanic glasses and melt inclusions reveal a carbonaceous chondrite heritage. Science, **340,** 1317-1320 (2013).
72. Arndt, J., Engelhardt, W.V., Gonzalez Cabeza, L., Meier, B. Formation of Apollo 15 green glass beads. *J. Geophys. Res.* **89,** C225-C232 (1984).
73. Cuk, M., Stewart, S. T. Making the Moon from a Fast-Spinning Earth: A Giant Impact Followed by Resonant Despinning. *Science* **338,** 1047-1052 (2012).
74. Warren, P. H. Stable-isotopic anomalies and the accretionary assemblage of the Earth and Mars: A subordinate role for carbonaceous chondrites. *Earth Plan Sci Lett*. **427**, 37-46 (2011)
75. Dauphas, N. *et al.* Geochemical arguments for an Earth-like Moon-forming impactor. *PNAS* **372**, 20130244 (2014).
76. Ringwood, A. E. Volatile and siderophile element geochemistry of the Moon: a reappraisal. *Earth Planet. Sci. Lett.* **111**, 537-555 (1992).
77. Warren, P. H. ''New'' lunar meteorites: implications for composition of the global lunar surface, lunar crust, and the bulk Moon. *Meteorit. Planet. Sci.* **40**, 477-506 (2005).
78. Warren, P. H., Dauphas, N. Revised estimation of the bulk composition of the Moon in light of GRAIL results, and why heat flow should be a top priority for future lunar missions. ***45th*** *LPSC* 2298 (2014).
79. Warren, P. H., Siegler, M. A. The Earth-like, and Wänke-like bulk compositions of the Moon. ***79th*** *Annual Meeting of the Meteoritical Society*. Abstr. #6399 (2016).
80. Sossi, P.A., Moynier, F. Chemical and isotopic kinship of iron in the Earth and Moon deduced from the lunar Mg-suite. *Earth Planet. Sci. Lett.* **471,** 125-135 (2017).
81. Walker, R. J. Siderophile elements in Tracing Planetary Formation and Evolution. *Geochemical Pers. Lett.* (2016).
82. Warren, P. H., Taylor, G. J., Keil, K., Shirley, D. N., Wasson, J. T. Petrology and chemistry of two ''large'' granite clasts from the moon. *Earth Planet. Sci. Lett.* **64**, 175-185 (1983).
83. Mysen, B. O., Virgo, D., Seifert, F. A. The structure of silicate melts: implications for chemical and physical properties of natural magmas. *Rev. Geophys. Space Phys*. **20**, 353–383 (1982).

Fig. S1. Backscattered electron image of a typical run product (LG10S-1) equilibrated at 1883 K and 1 GPa showing clear separation of metal and silicate. The silicate melt quenched to a mixture of glass and spinifex quench olivines. Crystals along the capsule walls are olivine and were excluded from analyses.

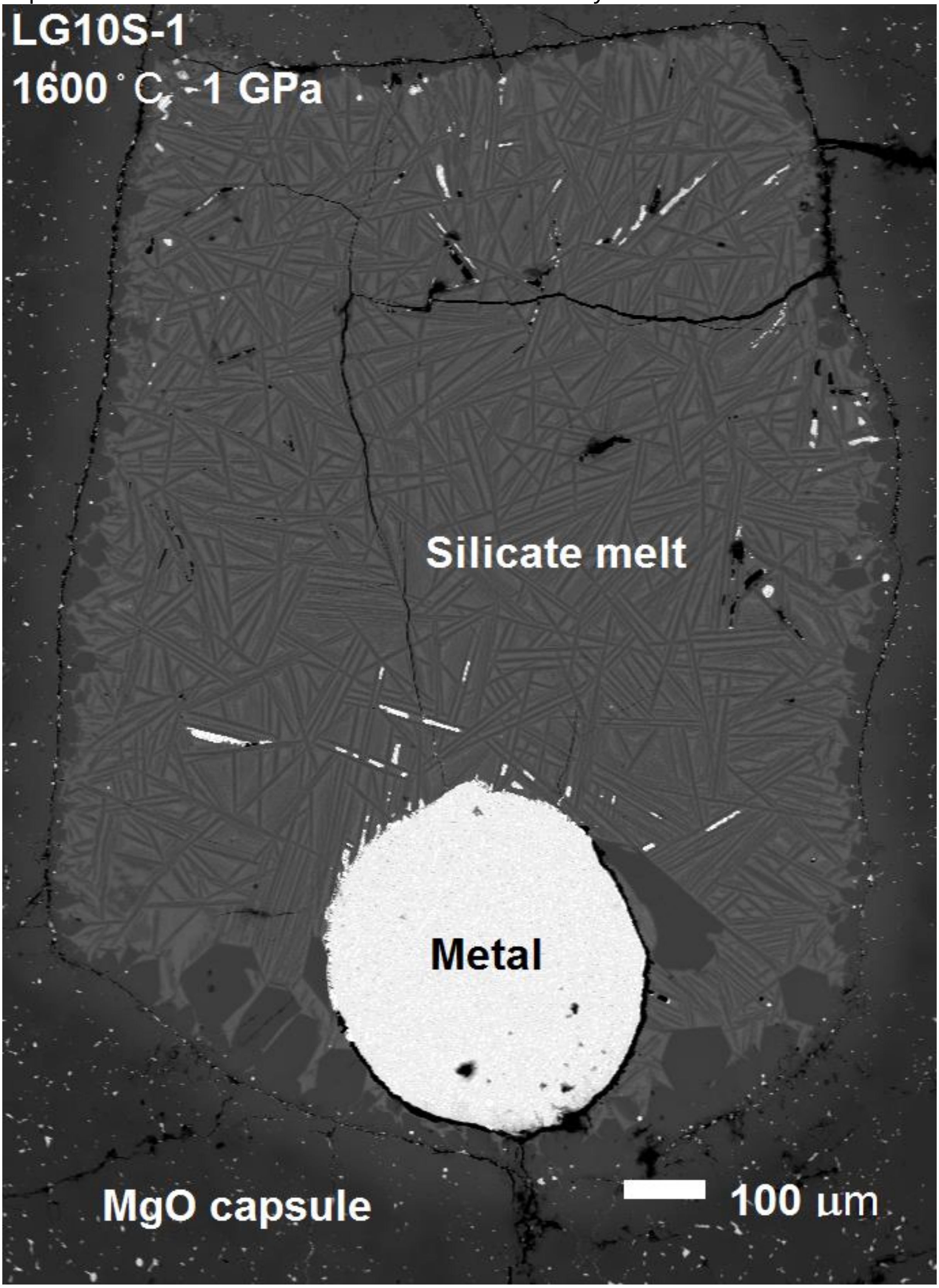

Fig. S2. Comparison between measured abundances of Ni, Pb, In, Mn, Cr, As, Cd, Sb and recommended values in silicate external standards for (a) BCR-2G and (b) BIR-1G. *N* is the amount of analyses (this study). Errors are 1SD. Lines are 1:1 identity lines plotted for reference. Recommended values (or published ranges) from GeoReM database version 18[54].

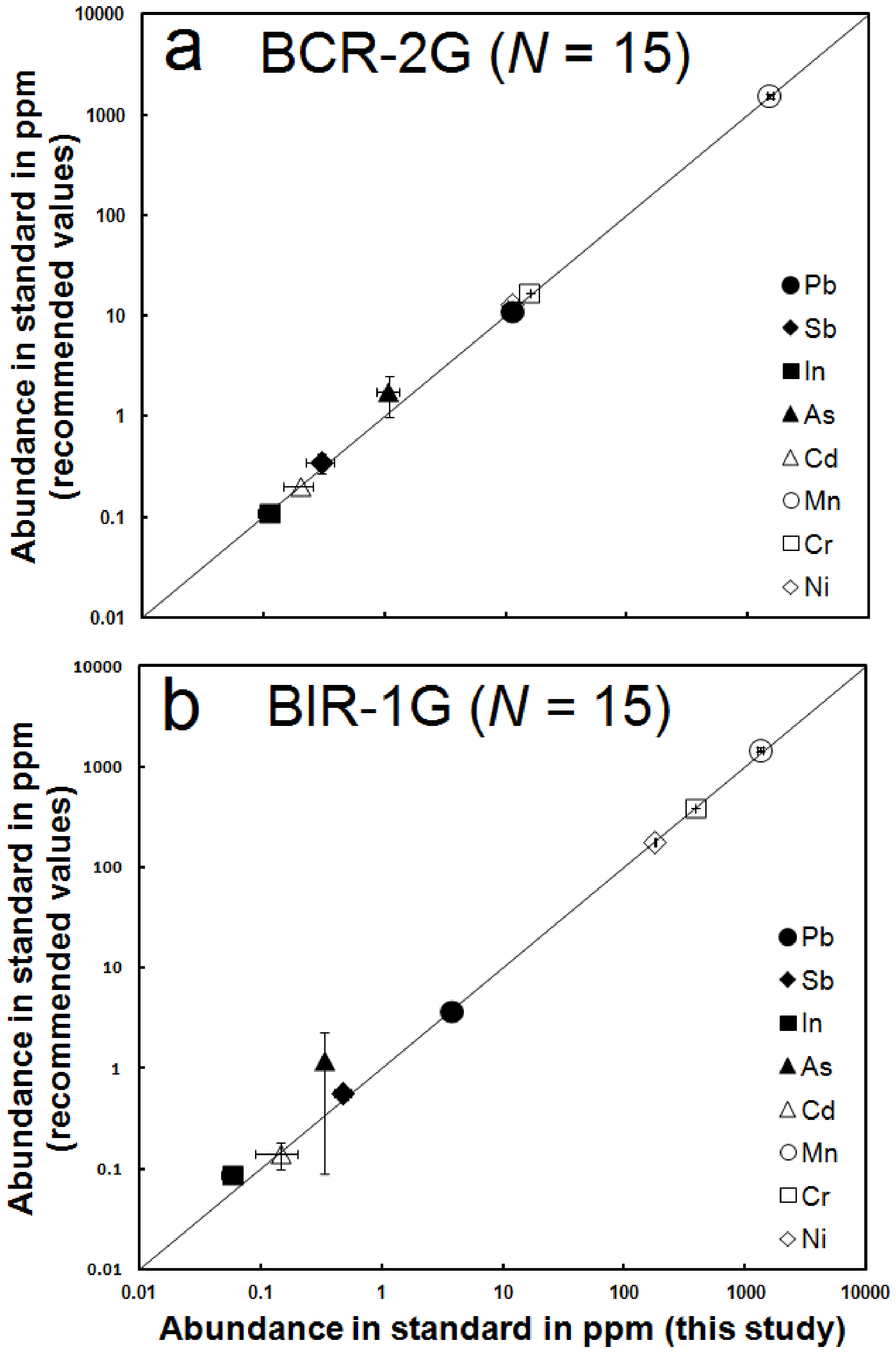

Fig. S3. Comparison between measured abundances of Pb, In, Mn, Cr, Cd, Se, Te in the silicate melt measured by LA-ICP-MS versus results obtained using electron microprobe (EMP). Errors for LA-ICP-MS are 1SE and errors for EMP are 2SE.

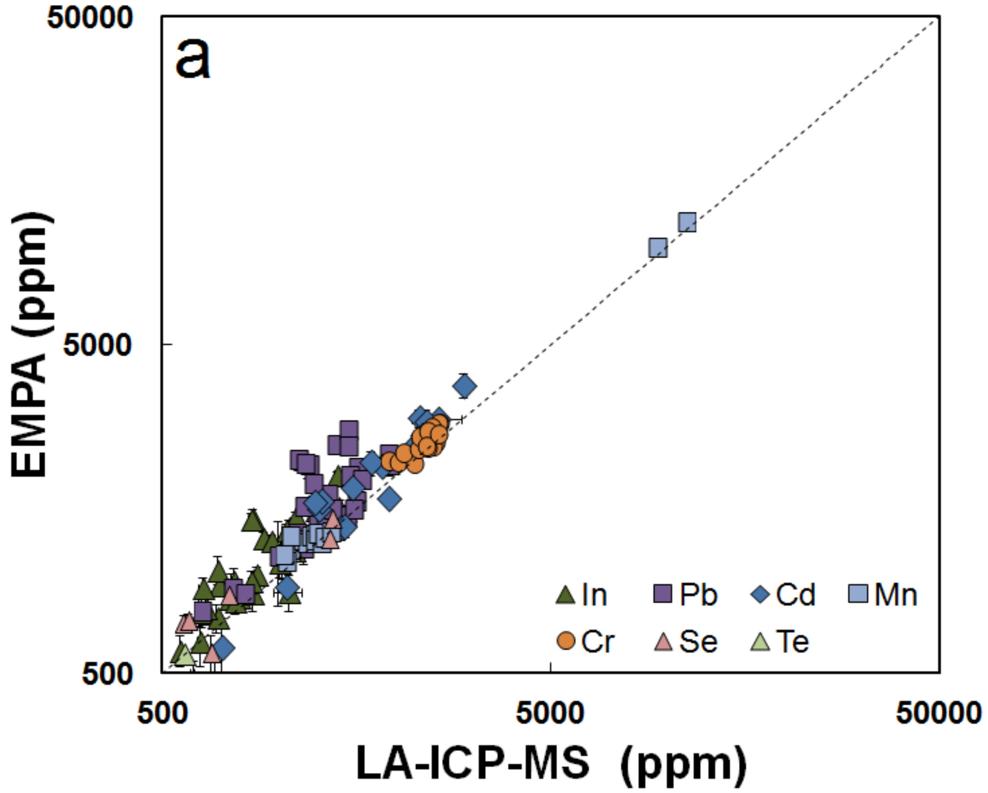

Fig. S4. Log D (S, Te, Se) at 1 GPa and 1783 K versus run time (in min). D's were corrected for different FeO according to the relationships provided in text. Vertical errors for log D (S, Se, Te) represent propagated errors assuming 1SE error for LA-ICP-MS and 2SE for EMP measurements.

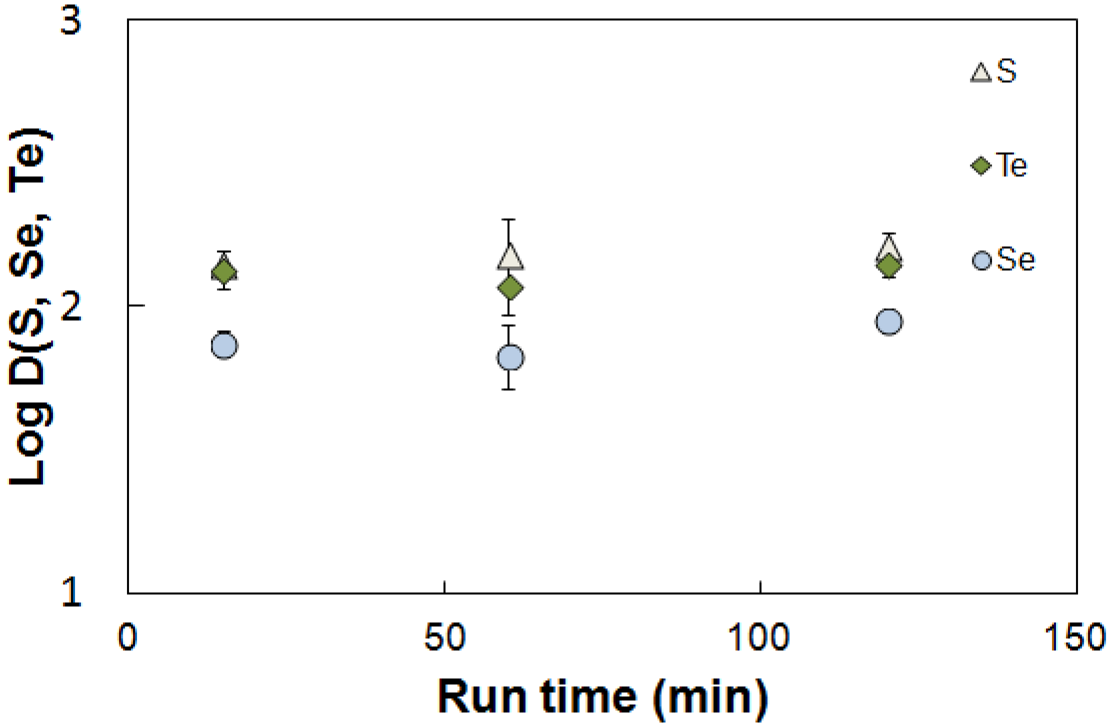

Figure S5. Effect of FeO of the silicate melt on uncorrected D(S, Se, Te) obtained at 1783 K and 1 GPa, at which the dependency with FeO is best quantified. Log D (Se, Te) are from low S (X_S < 0.04) experiments only. Lines are linear fits through data. Comparison data from refs (59,60). Vertical errors for log D (S, Se, Te) represent propagated errors assuming 1SE error for LA-ICP-MS and 2SE for EMP measurements. Vertical dashed line represents the estimated FeO molar fraction of the lunar mantle as summarized in ref (3).

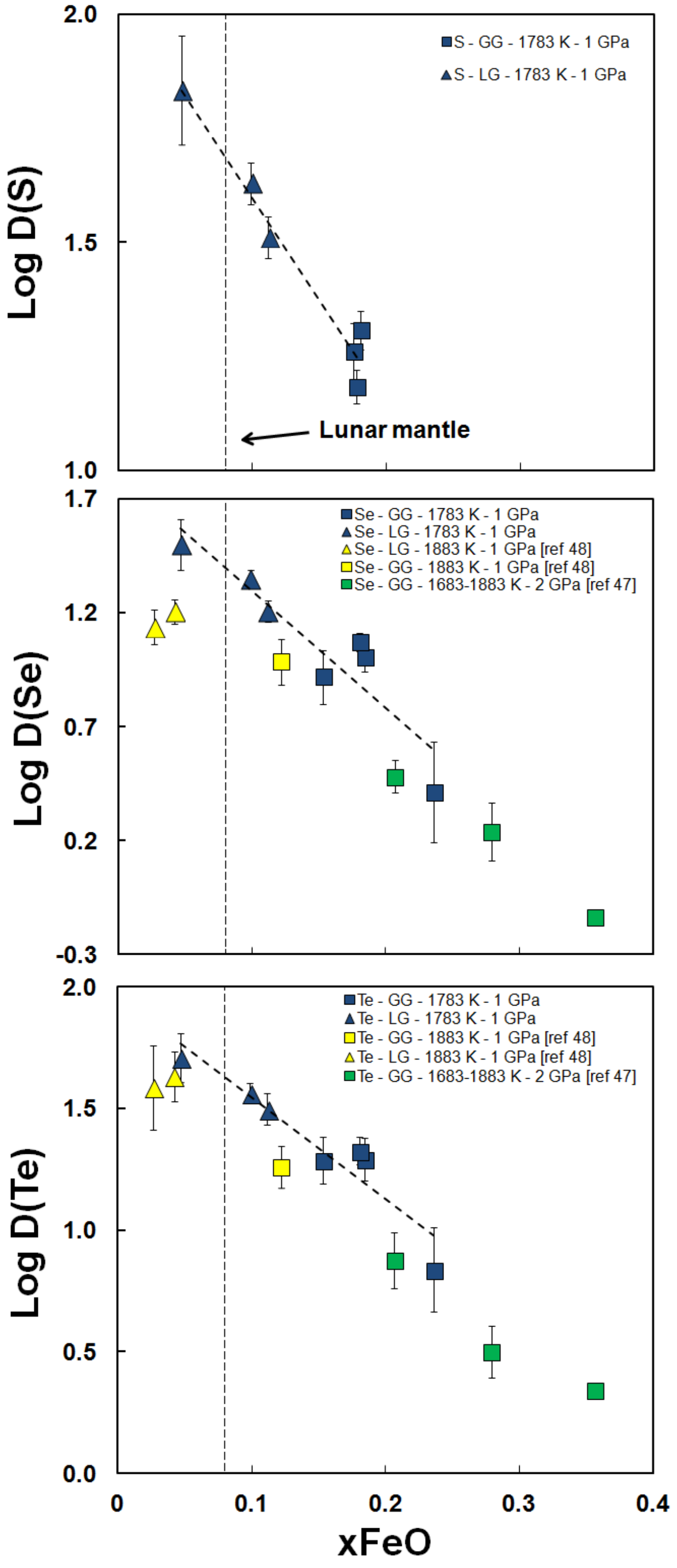

Figure S6. Effect of temperatures corrected D(S, Se, Te) values obtained from this study and Rose-Weston et al. (ref. 15). Log D (Se, Te) from this study are from low S (X_S < 0.04) experiments only. All log D values were corrected to a common reference pressure of 3 GPa and X_{FeO} = 0.08. Errors are usually smaller than symbol sizes.

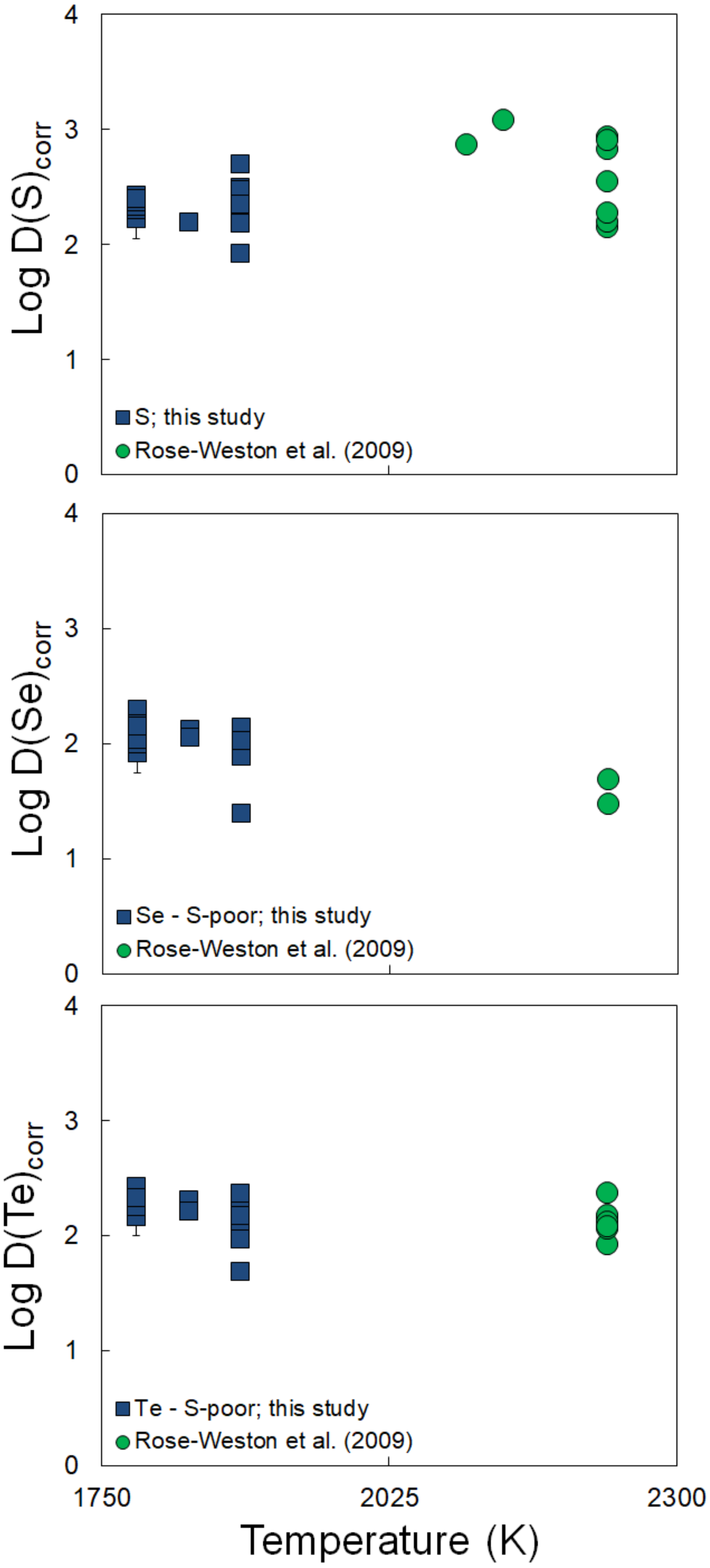

Table S1. Composition of the silicate starting compositions (1SD errors in brackets)

	N[a]	SiO_2	FeO	MgO	CaO	Al_2O_3	TiO_2	Cr_2O_3	MnO	K_2O	Na_2O	Total
**A-15 Green Glass (GG1)**[b]	15	48.1(3)	15.1(6)	19.5(4)	8.4(0)	7.9(1)	0.26(3)	0.55(2)	0.18(2)	-	-	100.0(5)
**A-15 Green Glass (GG2)**	17	48.3(1)	15.6(6)	17.2(4)	8.9(0)	7.3(0)	0.25(1)	0.53(1)	0.19(1)	-	-	98.3(1)
**Lunar granite (LG)**[c]	33	70.0(2)	2.2(0)	0.36(1)	1.8(0)	14.9(0)	0.36(1)	-	0.04(1)	8.0(1)	0.55(2)	98.3(1)

[a]Number of EMP measurements [b]ref. (66) [c]ref. (82).

Table S2. Metallic starting compositions (in wt%)

	Fe	S	Ni	Cu	Zn	Mn	Sn	In	Cd	Nb	Ta	Se	Te	As	Sb	Pb
**A**	88	10		1.5	1.5	1.5	1.5	1.5	1.5	1.5	1.5					
**B**	73-88	0-15	5.0					1.0	1.0			1.0	1.0	1.0	1.0	1.0
**C**	67	33	5.0					1.0	1.0			1.0	1.0	1.0	1.0	1.0

Table S3. Experimental conditions (all runs performed in MgO capsules)

Run	P (GPa)	T(K)	Run time (min)	Starting composition	ΔIW[a]	*nbo/t*[b]
GG0S-1	1.0	1783	10	GG2; B+0% S	-1.39	1.98
GG0S-2	1.0	1783	15	GG2; B+0% S	-1.19	2.24
GG0S-3	1.0	1783	20	GG2; B+0% S	-1.56	1.77
GG0S-4	2.0	1833	15	GG2; B+0% S	-1.37	1.86
GG0S-5	2.5	1883	15	GG2; B+0% S	-1.36	2.03
GG5S-1	1.0	1783	15	GG2; B+5% S	-1.38	1.96
GG5S-2	2.0	1783	15	GG2; B+5% S	-1.43	1.79
GG5S-3	2.5	1883	15	GG2; B+5% S	-1.41	2.04
GGS-14	1.0	1783	15	GG1; A+10% S	-1.43	2.52
GGS-15	2.0	1783	15	GG1; A+10% S	-1.18	2.41
GG10S-1	1.0	1783	15	GG2; B+10% S	-1.39	1.95
GG10S-2	2.0	1833	15	GG2;B+10% S	-1.33	2.00
GG10S-3	2.5	1883	15	GG2; B+10% S	-1.17	2.21
GG15S-1	1.0	1883	15	GG2; B+15% S	-1.54	2.54
GG15S-2	2.0	1883	15	GG2; B+15% S	-1.12	2.12
GG15S-3	2.5	1833	15	GG2; B+15% S	-1.12	2.04
GGFeS-1	1.0	1883	15	GG2; C+FeS	-1.40	2.22
GGFeS-2	2.0	1883	15	GG2; C+FeS	-1.38	1.85
GGFeS-3	2.5	1883	15	GG2; C+FeS	-1.31	1.99
LG0S-1	1.0	1883	30	LG; B+0% S	-1.37	2.17
LG0S-2	2.0	1883	30	LG; B+0% S	-1.49	1.70
LG0S-3	2.5	1883	30	LG; B+0% S	-1.61	1.41
LG5S-1	1.0	1783	15	LG;B+5% S	-1.82	1.08
LG5S-2	1.0	1783	60	LG;B+5% S	-2.57	0.68
LG5S-3	1.0	1783	120	LG;B+5% S	-1.91	1.10
LG5S-4	2.0	1883	15	LG;B+5% S	-1.42	1.56
LG5S-5	2.5	1883	15	LG;B+5% S	-1.20	1.88
LG10S-1	1.0	1883	30	LG;B+10% S	-1.58	1.77
LG10S-2	2.0	1883	30	LG;B+10% S	-1.31	1.60
LG10S-3	2.5	1883	30	LG;B+10% S	-1.45	1.53
LG15S-1	1.0	1883	60	LG;B+15% S	-1.68	1.66
LG15S-2	2.0	1883	130	LG;B+15% S	-1.82	1.36
LG15S-3	2.5	1883	30	LG;B+15% S	-1.41	1.57

[a] assuming ideal mixing behavior of Fe
[b] calculated using ref. (83)

Table S4. Silicate melt major and minor element composition of the silicate melt determined by EPMA and LA-ICP-MS

Run #	GGS-14	GGS-15	GG0S-1	GG0S-2	GG0S-3	GG0S-4	GG0S-5	GG5S-1	GG5S-2	GG5S-3	GG10S-1	GG10S-2	GG10S-3
**EPMA (wt%)**	*N = 45*[a]	*N = 43*	*N = 73*	*N = 29*	*N = 49*	*N = 49*	*N = 48*	*N = 64*	*N = 52*	*N = 46*	*N = 48*	*N = 47*	*N = 50*
**MgO**	18.20 (1.68)[b]	11.93 (1.08)	17.83 (0.95)	15.48 (1.56)	17.29 (0.47)	15.28 (1.53)	17.04 (1.92)	17.57 (1.05)	17.00 (0.66)	18.82(1.80)	17.22 (0.91)	15.86 (1.89)	17.05 (2.76)
**SiO_2**	36.46 (0.10)	35.61 (0.07)	42.99 (0.24)	39.22 (0.39)	45.35 (0.14)	42.13 (0.35)	40.97 (0.42)	42.59 (0.24)	45.33 (0.27)	42.93 (0.51)	42.71 (0.20)	39.84 (0.38)	39.24 (0.44)
**Al_2O_3**	7.16 (0.48)	7.95 (0.45)	6.70 (0.24)	6.17 (0.52)	7.05 (0.10)	7.87 (0.48)	7.49 (0.66)	7.47 (0.28)	6.91 (0.20)	6.30 (0.49)	7.31 (0.20)	8.27 (0.71)	7.50 (1.06)
**CaO**	8.41 (0.67)	8.18 (0.51)	7.44 (0.34)	7.18 (0.67)	8.36 (0.19)	8.89 (0.63)	8.46 (0.84)	8.10 (0.40)	7.30 (0.24)	7.22 (0.64)	8.09 (0.35)	9.10 (0.87)	8.12 (1.18)
**FeO**	20.79 (0.20)	26.42 (0.30)	22.19 (0.13)	27.43 (0.36)	18.37 (0.14)	21.89 (0.23)	22.53 (0.30)	21.80 (0.15)	20.66 (0.21)	21.22 (0.20)	21.66 (0.14)	22.43 (0.38)	26.25 (0.48)
**MnO**	1.28 (0.02)	1.49 (0.01)	0.15 (0.00)	0.15 (0.01)	0.17 (0.00)	0.16 (0.01)	0.16 (0.00)	0.16 (0.00)	0.14 (0.00)	0.15 (0.01)	0.17 (0.01)	0.17 (0.00)	0.16 (0.00)
**Cr_2O_3**	0.40 (0.01)	0.41 (0.01)	0.33 (0.01)	0.32 (0.01)	0.40 (0.01)	0.42 (0.01)	0.39 (0.01)	0.37 (0.01)	0.32 (0.00)	0.35 (0.01)	0.41 (0.01)	0.38 (0.01)	0.36 (0.01)
**TiO_2**	0.32 (0.02)	0.24 (0.01)	0.23 (0.01)	0.22 (0.02)	0.25 (0.01)	0.27 (0.02)	0.26 (0.02)	0.27 (0.01)	0.24 (0.01)	0.22 (0.02)	0.25 (0.01)	0.28 (0.03)	0.23 (0.03)
**SO_3**	0.61 (0.05)	0.59 (0.04)	0.01 (0.00)	0.01 (0.00)	0.01 (0.00)	0.01 (0.00)	0.01 (0.00)	0.22 (0.01)	0.11 (0.01)	0.08 (0.01)	0.34 (0.01)	0.09 (0.01)	0.27 (0.04)
**CuO**	0.03 (0.01)	0.03 (0.01)											
**ZnO**	1.49 (0.07)	1.60 (0.02)											
**SeO2**			0.12 (0.01)	0.18 (0.02)	0.08 (0.01)	0.10 (0.01)	0.07 (0.01)	0.06 (0.01)	0.03 (0.01)	0.02 (0.01)	0.03 (0.00)	0.00 (0.00)	0.01 (0.00)
**Nb_2O_5**	1.73 (0.14)	2.36 (0.14)											
**CdO**	0.16 (0.01)	0.08 (0.01)	0.34 (0.02)	0.43 (0.04)	0.27 (0.01)	0.34 (0.02)	0.33 (0.03)	0.21 (0.01)	0.24 (0.01)	0.25 (0.02)	0.18 (0.01)	0.19 (0.02)	0.29 (0.02)
**In_2O_3**	0.14 (0.01)	0.18 (0.01)	0.10 (0.01)	0.11 (0.01)	0.12 (0.01)	0.12 (0.01)	0.10 (0.01)	0.11 (0.01)	0.11 (0.01)	0.09 (0.01)	0.16 (0.01)	0.09 (0.01)	0.08 (0.01)
**SnO_2**	0.01 (0.01)	0.00 (0.00)											
**TeO_2**			0.03 (0.01)	0.05 (0.01)	0.01 (0.00)	0.01 (0.00)	0.01 (0.01)	0.02 (0.00)	0.01 (0.00)	0.01 (0.01)	0.01 (0.00)	0.01 (0.00)	0.01 (0.00)
**Ta_2O_5**	1.29 (0.10)	1.68 (0.09)											
**PbO**			0.17 (0.01)	0.16 (0.02)	0.16 (0.01)	0.19 (0.02)	0.16 (0.02)	0.12 (0.01)	0.16 (0.01)	0.14 (0.02)	0.17 (0.01)	0.12 (0.02)	0.10 (0.02)
**Total**	98.57 (0.23)	98.77 (0.13)	98.81 (0.15)	97.24 (0.26)	98.04 (0.16)	97.80 (0.20)	98.12 (0.22)	99.20 (0.14)	98.73 (0.14)	98.00 (0.23)	98.84 (0.15)	96.96 (0.25)	99.92 (0.25)
**LA-ICP-MS (ppm)**	*N = 10*	*N = 6*	*N = 2*	*N = 2*	*N = 5*	*N = 10*	*N = 5*	*N = 6*	*N = 9*	*N = 3*	*N = 9*	*N = 10*	*N = 5*
**Ca43**	64580 (1034)[b]	57293 (606)	53373 (2168)	49346 (22)	57583 (451)	53967 (654)	52590 (1091)	54971 (751)	49504 (390)	46055 (1889)	51962 (1121)	56069 (1205)	54173 (1158)
**Ti47**	1466 (20)	1464 (15)	1294 (37)	1156 (5)	1326 (14)	1306 (15)	1249 (18)	1285 (13)	1171 (9)	1127 (48)	1297 (42)	1396 (30)	1290 (30)
**Cr53**	2405 (16)	2581 (15)	1912 (92)	2020 (1)	2450 (16)	2560 (40)	2319 (19)	2507 (9)	2207 (22)	2076 (22)	2459 (66)	2323 (19)	2468 (22)
**Mn55**	9324 (39)	11057 (48)	1062 (38)	1062 (19)	1238 (10)	1149 (6)	1138 (10)	1169 (4)	1037 (4)	1022 (4)	1193 (32)	1218 (9)	1224 (8)
**Ni60**			38 (5)	49 (3)	15 (1)	40 (1)	52 (2)	37 (2)	29 (1)	50 (4)	45 (1)	48 (1)	75 (2)
**Cu63**	145 (8)	165 (5)											
**Zn66**	12974 (135)	13089 (157)											
**As76**			b.d.l [c]	b.d.l.	0.3 (0.0)	0.4 (0.1)	b.d.l.	1.3 (0.2)	0.7 (0.2)	0.6 (0.2)	0.2 (0.0)	0.4 (0.1)	b.d.l.
**Se82**			737 (9)	1338 (125)	666 (7)	564 (6)	597 (8)	416 (4)	237 (8)	203 (7)	256 (6)	143 (4)	189 (3)
**Nb93**	12483 (217)	16327 (170)											
**Cd111**	1433 (14)	1039 (12)	2559 (373)	2967 (23)	2159 (30)	2286 (75)	2380 (63)	1533 (11)	1831 (16)	1715 (65)	1255 (39)	1277 (37)	1228 (13)
**In115**	1083 (11)	1416 (26)	740 (10)	1054 (91)	868 (12)	761 (9)	785 (17)	694 (5)	774 (16)	620 (16)	1113 (25)	688 (17)	623 (8)
**Sn118**	191 (13)	280 (9)											
**Sb121**			b.d.l.	b.d.l.	1.0 (0.2)	1.3 (0.1)	1.1 (0.1)	1.3 (0.2)	0.5 (0.0)	1.4 (0.2)	0.8 (0.0)	0.8 (0.1)	0.8 (0.0)
**Te125**			347 (8)	492 (44)	264 (5)	198 (2)	200 (3)	182 (3)	135 (3)	109 (1)	226 (10)	164 (6)	86 (3)
**Ta181**	10309 (187)	14154 (163)											
**Pb208**			1573 (64)	1240 (11)	1424 (11)	1324 (31)	1310 (31)	1010 (15)	1484 (38)	1100 (29)	1359 (39)	991 (24)	751 (12)

Run #	GG15S-1	GG15S-2	GG15S3	LG0S-1	LG0S-2	LGOS-3	LG5S-1	LG5S-2	LG5S-3	LG5S-4	LG5S-5	LG10S-1	LG10S-2
**EPMA (wt%)**	*N = 32*	*N = 47*	*N = 62*	*N = 62*	*N = 49*	*N = 56*	*N = 75*	*N = 42*	*N = 62*	*N = 49*	*N = 37*	*N = 58*	*N = 47*
**MgO**	25.03 (2.99)	17.13 (1.69)	16.38 (1.99)	22.06 (1.02)	18.41 (1.33)	17.11 (0.53)	15.32 (0.03)	12.78 (0.13)	16.70 (0.02)	16.93 (0.68)	16.44 (1.49)	22.28 (0.74)	17.33 (0.64)
**SiO_2**	38.25 (0.19)	40.36 (0.23)	42.04 (0.63)	38.34 (0.05)	41.74 (0.33)	46.24 (0.15)	53.00 (0.06)	60.03 (0.19)	52.09 (0.06)	45.16 (0.15)	40.01 (0.22)	43.14 (0.10)	43.37 (0.11)
**Al_2O_3**	7.22 (0.89)	7.88 (0.51)	6.78 (0.70)	8.52 (0.34)	9.58 (0.47)	9.60 (0.19)	9.35 (0.01)	10.77 (0.03)	10.16 (0.02)	8.62 (0.17)	8.51 (0.51)	9.21 (0.22)	9.69 (0.18)
**CaO**	8.38 (1.16)	8.87 (0.67)	7.39 (0.81)	1.03 (0.05)	1.22 (0.10)	1.20 (0.04)	1.16 (0.01)	1.24 (0.01)	1.29 (0.00)	1.02 (0.05)	0.99 (0.11)	1.05 (0.05)	1.07 (0.05)
**FeO**	18.62 (0.59)	23.83 (0.28)	24.99 (0.44)	23.00 (0.35)	19.29 (0.36)	16.70 (0.18)	13.16 (0.03)	5.41 (0.16)	11.80 (0.02)	20.33 (0.26)	25.66 (0.38)	16.91 (0.29)	20.47 (0.34)
**Na_2O**				0.43 (0.02)	0.53 (0.03)	0.56 (0.01)	0.57 (0.01)	0.61 (0.01)	0.64 (0.01)	0.50 (0.01)	0.29 (0.02)	0.47 (0.02)	0.52 (0.01)
**K_2O**				5.10 (0.22)	5.93 (0.29)	6.27 (0.11)	6.28 (0.01)	7.32 (0.06)	7.18 (0.01)	5.46 (0.10)	5.29 (0.31)	5.41 (0.13)	5.87 (0.10)
**MnO**	0.16 (0.01)	0.17 (0.01)	0.17 (0.00)	0.02 (0.00)	0.02 (0.00)	0.02 (0.00)	0.03 (0.00)	0.02 (0.00)	0.03 (0.00)	0.03 (0.00)	0.03 (0.00)	0.04 (0.00)	0.04 (0.00)
**Cr_2O_3**	0.35 (0.01)	0.36 (0.01)	0.39 (0.01)										
**TiO_2**	0.21 (0.03)	0.24 (0.02)	0.20 (0.02)	0.24 (0.01)	0.26 (0.02)	0.27 (0.01)	0.26 (0.00)	0.28 (0.00)	0.28 (0.00)	0.24 (0.01)	0.23 (0.02)	0.26 (0.01)	0.26 (0.00)
**SO_3**	0.37 (0.05)	0.73 (0.15)	0.44 (0.05)	0.01 (0.00)	0.01 (0.00)	0.01 (0.00)	0.09 (0.00)	0.04 (0.00)	0.11 (0.00)	0.14 (0.01)	0.17 (0.02)	0.24 (0.01)	0.35 (0.02)

**SeO2**	0.01 (0.01)	0.01 (0.01)	0.01 (0.00)	0.21 (0.01)	0.10 (0.01)	0.06 (0.01)	0.03 (0.00)	0.00 (0.00)	0.02 (0.00)	0.03 (0.01)	0.04 (0.01)	0.02 (0.01)	0.01 (0.00)
**CdO**	0.00 (0.00)	0.06 (0.01)	0.05 (0.03)	0.16 (0.01)	0.30 (0.02)	0.19 (0.01)	0.00 (0.00)	0.01 (0.00)	0.15 (0.01)	0.05 (0.01)	n.d.	0.02 (0.00)	0.03 (0.01)
**In_2O_3**	0.11 (0.01)	0.07 (0.01)	0.13 (0.01)	0.17 (0.01)	0.18 (0.01)	0.18 (0.01)	0.20 (0.00)	0.24 (0.01)	0.18 (0.00)	0.19 (0.01)	0.12 (0.01)	0.16 (0.01)	0.14 (0.01)
**TeO_2**	0.02 (0.01)	0.01 (0.01)	0.01 (0.00)	0.07 (0.01)	0.03 (0.01)	0.03 (0.01)	0.03 (0.00)	0.01 (0.00)	0.01 (0.00)	0.03 (0.01)	0.02 (0.01)	0.04 (0.01)	0.01 (0.00)
**PbO**	0.23 (0.04)	0.08 (0.01)	0.17 (0.02)	0.27 (0.02)	0.23 (0.02)	0.22 (0.01)	0.25 (0.01)	0.13 (0.01)	0.21 (0.01)	0.23 (0.01)	0.24 (0.03)	0.30 (0.02)	0.20 (0.02)
**Total**	99.26 (0.31)	100.09 (0.17)	99.84 (0.15)	99.96 (0.08)	98.16 (0.25)	99.00 (0.10)	100.02 (0.06)	99.21 (0.15)	101.14 (0.08)	99.29 (0.10)	98.31 (0.16)	99.86 (0.11)	99.65 (0.12)
**LA-ICP-MS (ppm)**	*N = 7*	*N = 11*	*N = 11*	*N = 8*	*N = 10*	*N = 8*	*N = 5*	*N = 3*	*N = 10*	*N =*	*N = 2*	*N =7*	*N = 9*
**Ca43**	58589 (1244)	59771 (628)	45588 (482)	6735 (65)	7324 (60)	7482 (79)	8169 (149)	9862 (522)	9070 (50)	6726 (108)	6077 (76)	6485 (65)	7172 (75)
**Ti47**	1295 (17)	1465 (13)	1090 (12)	1398 (8)	1423 (11)	1487 (9)	1621 (19)	1835 (61)	1665 (11)	1389 (24)	1292 (4)	1446 (8)	1557 (15)
**Cr53**	2260 (27)	2393 (9)	2295 (16)										
**Mn55**	1267 (12)	1312 (5)	1061 (5)	132 (0)	130 (1)	148 (1)	254 (5)	223 (12)	216 (1)	216 (2)	206 (2)	256 (2)	295 (1)
**Ni60**	59 (2)	57 (1)	69 (1)	47 (0)	36 (1)	38 (1)	24 (2)	var.	15 (1)	38 (1)	50 (1)	38 (1)	51 (1)
**As76**	0.4 (0.1)	0.3 (0.0)	0.2 (0.0)	0.2 (0.1)	b.d.l.	0.3 (0.0)	1.4 (0.2)	b.d.l.	0.3 (0.0)	0.5 (0.1)	b.d.l.	b.d.l.	b.d.l.
**Se82**	233 (5)	161 (2)	143 (1)	1361 (16)	578 (8)	369 (5)	248 (7)	100 (2)	241 (4)	309 (6)	367 (7)	248 (2)	196 (3)
**Cd111**	1061 (32)	720 (11)	923 (17)	1470 (20)	2317 (50)	1906 (42)	955 (22)	254 (9)	424 (3)	732 (68)	404 (0)	422 (6)	580 (6)
**In115**	848 (15)	552 (5)	996 (10)	1055 (11)	850 (11)	842 (9)	1264 (15)	1401 (22)	1098 (6)	1232 (12)	692 (3)	1056 (6)	1008 (27)
**Sb121**	1.1 (0.1)	1.1 (0.0)	1.0 (0.1)	1.8 (0.1)	1.2 (0.1)	1.3 (0.1)	1.9 (0.2)	b.d.l.	0.3 (0.0)	0.9 (0.1)	0.8 (0.0)	0.5 (0.0)	0.5 (0.0)
**Te125**	322 (8)	78 (3)	90 (1)	568 (6)	216 (4)	127 (2)	163 (5)	63 (5)	134 (4)	165 (2)	197 (5)	340 (4)	111 (11)
**Pb208**	1182 (22)	630 (6)	1150 (18)	1387 (11)	1575 (25)	1510 (19)	1893 (20)	1148 (32)	1614 (15)	1919 (31)	1114 (10)	1498 (15)	1217 (37)
**Run #**	**LG10S-3**	**LG15S-1**	**LG15S-2**	**LG15S-3**	**GGFeS-1**	**GGFeS-2**	**GGFeS-3**						
**EPMA (wt%)**	*N = 45*	*N = 49*	*N = 55*	*N = 58*	*N = 41*	*N = 34*	*N = 36*						
**MgO**	18.26 (0.75)	22.19 (0.58)	20.06 (0.37)	21.63 (1.62)	26.25 (2.53)	22.28 (1.32)	23.46 (2.09)						
**SiO_2**	45.80 (0.16)	44.48 (0.09)	48.61 (0.10)	37.29 (0.15)	42.97 (0.38)	47.21 (0.36)	45.07 (0.5)						
**Al_2O_3**	9.04 (0.21)	9.54 (0.15)	9.71 (0.11)	15.72 (0.92)	7.36 (0.63)	7.15 (0.26)	7.38 (0.55)						
**CaO**	1.02 (0.05)	1.16 (0.04)	1.16 (0.04)	1.70 (0.12)	8.57 (0.90)	8.40 (0.44)	8.50 (0.78)						
**FeO**	18.60 (0.27)	14.33 (0.24)	11.80 (0.15)	18.80 (0.21)	12.86 (0.42)	12.77 (0.24)	13.74 (0.13)						
**Na_2O**	0.49 (0.02)	0.49 (0.01)	0.53 (0.01)	0.40 (0.03)									
**K_2O**	5.50 (0.11)	5.81 (0.10)	6.05 (0.07)	4.07 (0.24)									
**MnO**	0.03 (0.00)	0.03 (0.00)	0.03 (0.00)	0.03 (0.00)	0.17 (0.01)	0.17 (0.01)	0.17 (0.01)						
**Cr_2O_3**	0.01 (0.00)	0.00 (0.00)	0.00 (0.00)	0.00 (0.00)	0.40 (0.01)	0.36 (0.01)	0.39 (0.01)						
**TiO_2**	0.24 (0.01)	0.26 (0.01)	0.26 (0.01)	0.18 (0.01)	0.23 (0.02)	0.22 (0.01)	0.22 (0.02)						
**SO_3**	0.19 (0.01)	0.41 (0.02)	0.16 (0.01)	0.24 (0.02)	0.93 (0.19)	0.95 (0.20)	0.69 (0.10)						
**SeO2**	0.00 (0.00)	0.00 (0.00)	0.00 (0.00)	0.00 (0.00)	0.00 (0.00)	0.00 (0.00)	0.00 (0.00)						
**CdO**	0.05 (0.01)	0.01 (0.00)	0.01 (0.01)	0.00 (0.00)	0.07 (0.02)	0.06 (0.01)	0.03 (0.01)						
**In_2O_3**	0.17 (0.01)	0.16 (0.01)	0.15 (0.01)	0.11 (0.01)	0.10 (0.01)	0.12 (0.01)	0.10 (0.01)						
**TeO_2**	0.02 (0.01)	0.02 (0.00)	0.01 (0.00)	0.02 (0.00)	0.00 (0.00)	0.00 (0.00)	0.01 (0.00)						
**PbO**	0.17 (0.01)	0.23 (0.02)	0.27 (0.01)	0.10 (0.01)	0.03 (0.01)	0.03 (0.01)	0.02 (0.01)						
**Total**	100.00 (0.10)	99.46 (0.12)	99.13 (0.06)	100.31 (0.15)	100.06 (0.29)	100.09 (0.16)	100.12 (0.15)						
**Run #**	**LG10S-3**	**LG15S-1**	**LG15S-2**	**LG15S-3**	**GGFeS-1**	**GGFeS-2**	**GGFeS-3**						
**LA-ICP-MS (ppm)**	*N = 12*	*N = 8*	*N = 5*	*N = 4*	*N =8*	*N = 10*	*N = 11*						
**Ca43**	6526 (168)	7196 (80)	8123 (222)	11065 (504)	56058 (1234)	55070 (828)	59750 (859)						
**Ti47**	1414 (34)	1480 (8)	1625 (21)	1084 (44)	1320 (38)	1299 (20)	1377 (19)						
**Cr53**					2401 (27)	2389 (18)	2550 (22)						
**Mn55**	250 (4)	251 (3)	254 (4)	234 (4)	1246 (9)	1291 (13)	1340 (9)						
**Ni60**	46 (1)	46 (1)	39 (3)	66 (2)	44 (3)	57 (2)	77 (4)						
**As76**	0.7 (0.0)	0.5 (0.2)	0.7 (0.1)	0.7 (0.1)	9 (1)	36 (1)	11 (1)						
**Se82**	140 (4)	168 (1)	81 (5)	106 (4)	117 (2)	45 (1)	48 (1)						
**Cd111**	707 (24)	236 (5)	266 (8)	48 (3)	710 (22)	682 (19)	432 (10)						
**In115**	1036 (23)	905 (6)	952 (23)	632 (15)	644 (9)	840 (10)	765 (14)						
**Sb121**	0.5 (0.1)	1.4 (0.1)	1.6 (1.0)	2.7 (0.0)	28 (2)	41 (2)	37 (3)						
**Te125**	89 (2)	142 (3)	57 (3)	59 (1)	46 (2)	19 (1)	10 (1)						
**Pb208**	1547 (27)	1159 (12)	1493 (65)	808 (33)	210 (7)	226 (3)	124 (5)						

[a] Number of analyses [b] Values in parentheses are two standard errors for EPMA and one standard error for LA-ICP-MS in terms of least digits cited [c]b.d.l. = below detection limit.

Table S5. Metallic melt major and minor element composition of the metallic melt determined by EPMA and LA-ICP-MS

**Run #**	**GGS-14**	**GGS-15**	**GG0S-1**	**GG0S-2**	**GG0S-3**	**GG0S-4**	**GG0S-5**	**GG5S-1**	**GG5S-2**	**GG5S-3**	**GG10S-1**	**GGS10S-2**	**GG10S-3**
**EPMA (wt%)**	*N = 45*[a]	*N = 43*	*N = 49*	*N = 35*	*N = 36*	*N = 48*	*N =49*	*N = 64*	*N = 52*	*N = 34*	*N = 27*	*N = 49*	*N = 61*
**Fe**	92.07 (0.22)[b]	92.08 (0.23)	87.50 (0.53)	87.71 (0.77)	85.83 (0.59)	84.24 (0.43)	84.51 (0.46)	87.89 (0.26)	87.70 (0.25)	83.72 (0.56)	82.98 (0.48)	84.41 (0.33)	84.34 (0.46)
**Cr**	0.03 (0.00)	0.02 (0.00)	0.03 (0.02)	0.01 (0.00)	0.03 (0.01)	0.02 (0.00)	0.02 (0.00)	0.02 (0.01)	0.01 (0.00)	0.03 (0.02)	0.03 (0.00)	0.02 (0.00)	0.00 (0.00)
**Mn**	0.00 (0.00)	0.00 (0.00)	0.00 (0.00)	0.00 (0.00)	0.00 (0.00)	0.00 (0.00)	0.00 (0.00)	0.00 (0.00)	0.00 (0.00)	0.00 (0.00)	0.00 (0.00)	0.00 (0.00)	0.00 (0.00)
**Ni**			5.90 (0.03)	5.77 (0.13)	5.36 (0.03)	6.09 (0.03)	5.96 (0.04)	5.92 (0.03)	5.58 (0.04)	5.72 (0.04)	6.76 (0.14)	7.03 (0.03)	7.31 (0.10)
**Cu**	0.59 (0.02)	0.66 (0.02)											
**Zn**	0.44 (0.05)	0.37 (0.04)											
**As**			0.79 (0.03)	0.61 (0.11)	0.73 (0.08)	0.80 (0.02)	0.78 (0.02)	1.45 (0.03)	1.20 (0.02)	1.26 (0.06)	1.32 (0.09)	1.41 (0.05)	1.23 (0.04)
**Se**			0.74 (0.11)	0.35 (0.16)	0.55 (0.15)	0.95 (0.16)	0.88 (0.17)	0.49 (0.04)	0.52 (0.04)	0.60 (0.08)	0.23 (0.04)	0.19 (0.03)	0.43 (0.04)
**Cd**[c]	0.17 (0.03)	0.14 (0.01)	0.27 (0.12)	0.46 (0.14)	0.29 (0.06)	0.21 (0.05)	0.24 (0.07)	0.21 (0.04)	0.23 (0.04)	0.28 (0.05)	0.14 (0.02)	0.20 (0.03)	0.21 (0.03)
**In**	0.56 (0.05)	0.88 (0.06)	0.48 (0.06)	0.20 (0.08)	0.48 (0.11)	0.66 (0.08)	0.66 (0.09)	0.31 (0.03)	0.43 (0.03)	0.54 (0.06)	0.34 (0.04)	0.47 (0.05)	0.47 (0.04)
**Sn**	1.05 (0.02)	1.29 (0.04)											
**Sb**			0.97 (0.07)	0.60 (0.21)	0.87 (0.17)	1.10 (0.08)	0.95 (0.06)	0.55 (0.04)	0.50 (0.02)	0.48 (0.06)	0.65 (0.09)	0.42 (0.05)	0.68 (0.05)
**Te**			0.68 (0.14)	0.34 (0.12)	0.51 (0.11)	0.66 (0.09)	0.72 (0.11)	0.39 (0.05)	0.55 (0.06)	0.62 (0.08)	0.40 (0.08)	0.42 (0.05)	0.45 (0.04)
**Pb**			0.36 (0.11)	0.19 (0.08)	0.21 (0.05)	0.24 (0.04)	0.38 (0.08)	0.18 (0.04)	0.21 (0.05)	0.39 (0.07)	0.09 (0.03)	0.20 (0.03)	0.30 (0.03)
**S**	3.85 (0.18)	3.55 (0.18)	0.09 (0.01)	0.02 (0.01)	0.08 (0.02)	0.14 (0.03)	0.09 (0.02)	1.79 (0.14)	1.59 (0.10)	1.50 (0.20)	2.49 (0.34)	0.80 (0.12)	3.75 (0.40)
**Total**	98.78 (0.09)	98.96 (0.11)	97.97 (0.10)	96.15 (0.14)	94.90(0.14)	95.18(0.11)	95.29 (0.13)	99.28 (0.08)	98.55 (0.09)	95.20 (0.17)	95.42 (0.12)	95.55 (0.11)	99.21 (0.10)

**Run #**	**GG15S-1**	**GG15S-2**	**GG15S-3**	**LG0S-1**	**LG0S-2**	**LGOS-3**	**LG5S-1**	**LG5S-2**	**LG5S-3**	**LG5S-4**	**LG5S-5**	**LG10S-1**	**LG10S-2**
**EPMA (wt%)**	*N = 56*	*N = 48*	*N = 58*	*N = 56*	*N = 55*	*N = 56*	*N = 71*	*N = 42*	*N = 70*	*N = 59*	*N = 57*	*N = 56*	*N = 56*
**Fe**	83.32 (0.13)	74.64 (0.85)	75.90 (2.38)	88.45 (0.76)	90.42 (0.23)	88.40 (0.59)	86.76 (0.31)	88.53 (0.76)	85.47 (0.35)	87.51 (0.27)	87.30 (0.39)	82.86 (0.30)	79.25 (0.32)
**Cr**	0.02 (0.00)	0.01 (0.00)	0.01 (0.00)								0.00 (0.00)		
**Mn**	0.00 (0.00)	0.00 (0.00)	0.00 (0.00)	0.00 (0.00)	0.00 (0.00)	0.00 (0.00)	0.00 (0.00)	0.00 (0.00)	0.00 (0.00)	0.00 (0.00)	0.00 (0.00)	0.00 (0.00)	0.00 (0.00)
**Ni**	8.28 (0.07)	5.51 (0.21)	8.43 (0.41)	5.77 (0.06)	5.92 (0.02)	6.08 (0.06)	5.36 (0.03)	6.15 (0.05)	5.70 (0.03)	5.83 (0.04)	6.16 (0.04)	7.12 (0.04)	6.25 (0.07)
**As**	1.52 (0.03)	0.66 (0.03)	1.99 (0.21)	0.41 (0.01)	0.42 (0.01)	0.44 (0.01)	0.91 (0.01)	1.26 (0.09)	0.99 (0.01)	1.04 (0.02)	0.98 (0.02)	1.34 (0.04)	0.88 (0.02)
**Se**	0.05 (0.01)	0.77 (0.05)	0.56 (0.13)	0.61 (0.19)	0.75 (0.07)	0.90 (0.13)	0.40 (0.04)	0.32 (0.08)	0.54 (0.04)	0.62 (0.04)	0.48 (0.05)	0.35 (0.02)	0.71 (0.02)
**Cd**	0.12 (0.00)	0.27 (0.04)	0.19 (0.07)	0.13 (0.11)	0.21 (0.03)	0.41 (0.10)	0.22 (0.05)	0.16 (0.03)	0.15 (0.00)	0.16 (0.01)	0.14 (0.00)	0.21 (0.01)	0.18 (0.02)
**In**	0.22 (0.01)	0.37 (0.04)	0.57 (0.14)	0.54 (0.07)	0.61 (0.04)	0.73 (0.06)	0.43 (0.04)	0.43 (0.08)	0.47 (0.04)	0.55 (0.04)	0.41 (0.03)	0.63 (0.03)	0.61 (0.03)
**Sb**	0.75 (0.03)	0.70 (0.06)	0.89 (0.18)	1.05 (0.02)	1.18 (0.02)	1.22 (0.03)	0.62 (0.02)	1.07 (0.15)	0.83 (0.03)	0.89 (0.05)	0.95 (0.05)	1.12 (0.05)	0.88 (0.04)
**Te**	0.17 (0.01)	0.62 (0.06)	0.97 (0.23)	0.49 (0.19)	0.40 (0.05)	0.78 (0.14)	0.51 (0.07)	0.32 (0.07)	0.49 (0.04)	0.72 (0.05)	0.50 (0.04)	0.95 (0.06)	0.65 (0.04)
**Pb**	0.05 (0.02)	0.55 (0.08)	0.28 (0.08)	0.60 (0.31)	0.18 (0.06)	0.66 (0.19)	0.51 (0.12)	0.29 (0.09)	0.37 (0.04)	0.46 (0.05)	0.48 (0.05)	1.10 (0.11)	0.85 (0.09)
**S**	1.30 (0.09)	12.88 (0.95)	7.46 (1.80)	0.05 (0.00)	0.04 (0.00)	0.10 (0.01)	1.14 (0.11)	1.04 (0.25)	1.87 (0.18)	1.74 (0.12)	1.93 (0.18)	2.31 (0.16)	7.43 (0.25)
**Total**	95.68 (0.15)	96.82 (0.11)	97.28 (0.33)	98.15 (0.10)	99.97 (0.09)	99.60 (0.43)	96.70 (0.16)	99.47 (0.18)	96.74 (0.21)	99.40 (0.11)	99.21 (0.12)	97.81 (0.09)	97.56 (0.13)

**Run #**	**LG10S-3**	**LG15S-1**	**LG15S-2**	**LG15S-3**	**GGFeS-1**	**GGFeS-2**	**GGFeS-3**
**EPMA (wt%)**	*N = 56*	*N = 44*	*N = 53*	*N = 56*	*N = 37*	*N = 20*	*N = 41*
**Fe**	82.01 (0.34)	79.58 (0.23)	77.91 (0.28)	78.80 (0.51)	60.10 (0.69)	60.22 (1.20)	58.40 (1.10)
**Cr**					0.06 (0.01)	0.27 (0.04)	0.16 (0.03)
**Mn**	0.00 (0.00)	0.00 (0.00)	0.00 (0.00)	0.00 (0.00)	0.03 (0.00)	0.04 (0.00)	0.02 (0.00)
**Ni**	6.69 (0.07)	8.92 (0.05)	7.42 (0.06)	8.88 (0.10)	1.61 (0.58)	1.24 (0.26)	3.35 (0.99)
**As**	1.10 (0.02)	1.72 (0.02)	1.36 (0.02)	1.74 (0.06)	0.13 (0.11)	0.07 (0.07)	0.40 (0.19)
**Se**	0.56 (0.03)	0.28 (0.02)	0.56 (0.02)	0.35 (0.03)	1.64 (0.08)	0.86 (0.04)	1.08 (0.10)
**Cd**	0.18 (0.02)	0.13 (0.00)	0.19 (0.00)	0.11 (0.00)	1.16 (0.49)	0.17 (0.17)	0.65 (0.43)
**In**	0.81 (0.05)	0.55 (0.02)	0.89 (0.05)	0.62 (0.05)	0.99 (0.30)	0.30 (0.19)	0.67 (0.28)
**Sb**	0.86 (0.04)	1.08 (0.05)	0.84 (0.03)	1.26 (0.07)	0.25 (0.10)	0.05 (0.03)	0.37 (0.17)
**Te**	0.88 (0.05)	0.72 (0.06)	1.09 (0.05)	0.84 (0.06)	0.90 (0.24)	0.45 (0.21)	0.77 (0.26)
**Pb**	0.68 (0.06)	0.75 (0.11)	1.47 (0.10)	0.49 (0.05)	0.41 (0.21)	0.16 (0.18)	0.06 (0.05)
**S**	4.44 (0.28)	3.77 (0.14)	5.49 (0.18)	4.41 (0.38)	31.54 (1.38)	34.15(0.56)	31.77 (1.12)
**Total**	98.09 (0.07)	97.38 (0.09)	97.02 (0.10)	97.42 (0.10)	98.30 (0.37)	98.03(0.41)	97.73 (0.34)

Table S6. Assessment of the maximum error on log D (S, Se, Te, Sb) due to low metal totals for some of the samples.

	Log D uncorrected	Log D corrected for lower metal totals
S	0.41±0.04*	0.44
Se	1.17±0.08	1.19
Te	1.56±0.07	1.58
Sb	3.93±0.03	3.96

***Errors were calculated by propagating 2SE error for EMP and 1SE for LA-ICP-MS measurements
